# A genetic model for development, physiology and behavior of catecholamine-deficient zebrafish larvae

**DOI:** 10.1038/s42003-026-10732-9

**Published:** 2026-08-05

**Authors:** Susana Paredes-Zúñiga, Rebecca Veit, Kristine Østevold, Gerard Arrey, Johanna Wehrle, Dennis Frank, Johannes Oswald, Florian Veit, Theresa Schredelseker, Sandra Glauben, Jochen Holzschuh, Wolfgang Driever

**Affiliations:** 1 Developmental Biology, Institute Biology 1, Faculty of Biology, University of Freiburg, Freiburg im Breisgau, Germany; 2 CIBSS and BIOSS - Centres for Biological Signalling Studies, University of Freiburg, Freiburg im Breisgau, Germany

**Keywords:** Genetics of the nervous system, Developmental neurogenesis

## Abstract

Dopamine and noradrenaline have conserved roles in control of physiology and behaviors of vertebrates. However, vertebrate genetic models completely devoid of catecholamines are not available. We generated genetically catecholamine-free zebrafish larvae by combining mutations in all three genes involved in L-DOPA biosynthesis: the *tyrosine hydroxylase* genes *th* and *th2*, and *tyrosinase tyr*. Here we show that catecholamine-deficient 5 day-old zebrafish larvae are viable and develop an anatomically normal nervous system, including the full complement of catecholaminergic neuron somata and projections. In contrast, selected physiological functions that depend on catecholamines are impaired, including hatching and heart rate regulation upon temperature challenges. Spontaneous locomotion and optomotor behaviors are also impaired. Despite the changes observed, it is surprising that larvae develop a largely normal behavioral repertoire. Our model will be useful to investigate the activities of additional neurotransmitters expressed in catecholaminergic neurons, and how physiology and neural circuit function are regulated in the absence of catecholamines.

## Abstract (web summary)

A genetic zebrafish model that is deficient for all catecholamines still displays normal development of catecholamine neuron somata and projections, but develops specific abnormalities in hatching, heart rate regulation, and motor behavior.

## Introduction

Catecholamines (CAs) are chemical messengers with neuromodulatory and endocrine functions. Endogenous CAs include dopamine (DA), noradrenaline (NA, norepinephrine), and adrenaline (epinephrine)^1–3^. In addition to the roles of CAs in physiology, progressive loss of DA neurons in Parkinson’s Disease^4^, hypodopaminergia, and altered DA signaling are some of the multiple causes behind neurodevelopmental disorders such as autism^5^ and attention deficit/hyperactivity disorder (ADHD)^6^.

CA neurons can be identified by the expression of the rate-limiting enzyme Tyrosine hydroxylase (TH), which catalyzes the hydroxylation of the amino acid L-tyrosine to L-dihydroxyphenylalanine (L-DOPA; Supplementary Fig. 1A). Therefore, the *Th* gene has become a target for genetic manipulation of CA biosynthesis. The disruption of the *Th* locus in mice leads to mid-gestational lethality^7,8^. However, surprisingly, *Th* mutant mice have been shown to still contain relevant amounts of DA in the brain and body^7^, which has been suggested to derive from maternal bloodstream^9^ and from melanocytes^10^. In melanocytes, the enzyme Tyrosinase (TYR) catalyzes the oxidation of tyrosine to dopaquinone, a precursor of melanin (Supplementary Fig. 1A)^11,12^. In redox reactions, dopaquinone is converted to L-DOPA^10^, which exits melanocytes and enters the bloodstream. In wild-type (WT) mice, Tyrosinase does not make a major contribution to DA levels in the brain^13^. However, in the absence of TH, Tyrosinase-derived L-DOPA partially substitutes NA and DA in peripheral tissues and the brain^13^. For these reasons, genetic mammalian models have so far not been used extensively to address the developmental and behavioral consequences of CA depletion.

Experimental models for DA depletion often use genetic or neurotoxin ablation of DA neurons, and pharmacological or optogenetic manipulation of DA signaling. However, these techniques do not selectively eliminate DA neuromodulation, but also non-DA functions of DA neurons, which typically have dual transmitter phenotypes, either GABAergic or glutamatergic^14–19^. Further, agonist or antagonist treatments broadly act on CA receptor-expressing cells, irrespective of physiological CA signaling, and are prone to cause gain-of-function and potentially indirect effects. Despite the advances in understanding CA systems, we have limited knowledge of the role of second transmitters in DA neurons, and of the modulatory functions of DA during brain development. Therefore, we aimed at developing a genetic zebrafish model completely devoid of catecholamine transmission, but still containing the cellular complement of wild-type CA neurons.

The CA systems in teleosts^20,21^, and specifically in zebrafish^22–25^, show both conserved and divergent features compared to mammals (Supplementary Fig. 1B and legend). While CA systems in the zebrafish adult brain have been extensively described, we will focus here on the experimentally more accessible larval stages^17,26–28^. In contrast to mammals, zebrafish have two TH-encoding genes, *th* (previously also called *th1*), and *th2*^29–32^. The *th2* gene likely got secondarily lost in mammals, since it is also found in amphibians and birds^29,32,33^. Many *th2* neurons co-express *th*^34,35^ (Supplementary Fig. 1B). The *tyrosinase (tyr)* locus has also been shown to contribute to physiologically relevant biosynthesis of L-DOPA in the retina^36^. Therefore, to generate a CA-free model in zebrafish, all three genes, *th*, *th2* and *tyr*, need to be inactivated.

Here, we generated *th*, *th2, tyr* triple mutant zebrafish larvae, validated the absence of DA by ELISA, and performed an initial analysis of embryonic and larval development in general, and of CA systems development in more detail. We also characterized potential effects of loss of CAs on cardiac function and basic optomotor behavior. *th*, *th2, tyr* triple mutant larvae develop largely normally up to 5 days post-fertilization (dpf), and are viable into larval stages. Mutant larvae, however, show distinct physiological and behavioral phenotypes affecting hatching, heartbeat regulation, and motor behaviors.

## Results

### Triple homozygous *th, th2, tyr* mutant larvae are devoid of dopamine

**Fig. 1:**
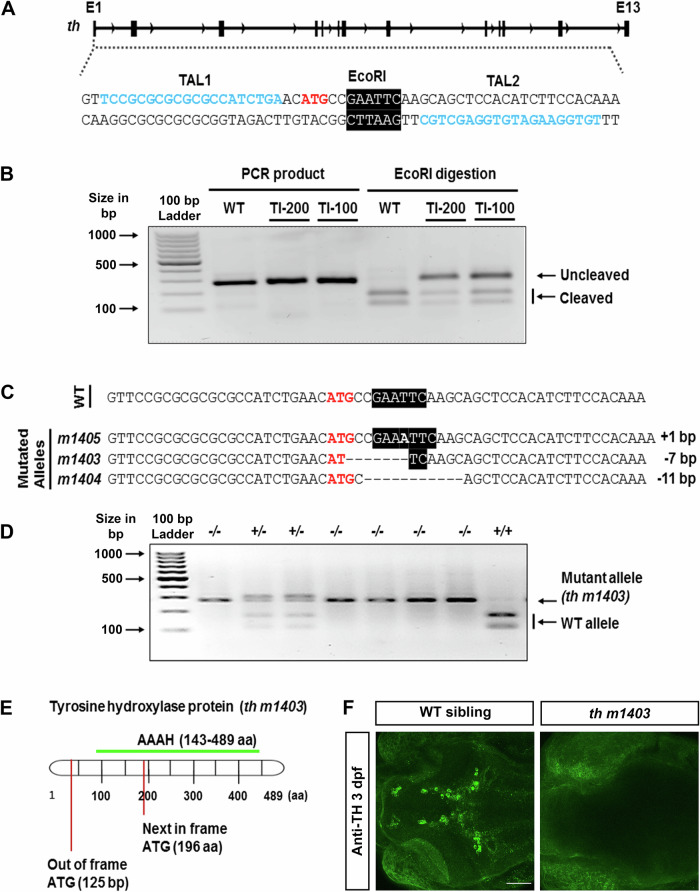
TALEN-mediated mutation of the *th* locus. **A** TALENs were designed to prevent Th translation by disrupting the beginning of the ORF. Schematic representation showing the binding sites for TALEN pairs (TAL1 and TAL2, blue) in exon 1 (E1). The start codon ATG is shown in red, and the recognition site for EcoRI is highlighted in black. **B** TALEN efficacy assay of injected G0 embryos. PCR amplification followed by EcoRI digestion shows that TALEN-injected embryos present an uncleaved band, indicating an indel mutation at the target site. TALEN-encoding mRNAs were injected at two different concentrations 100 ng/µl (TI-100) and 200 ng/µl (TI-200). WT: wild type, L: 100 bp ladder. **C** Sequencing results of the undigested bands from individual F1 heterozygous fish. WT refers to the reference wild-type allele. Mutated alleles: *m1405* (+1 bp addition), *m1403* (−7 bp deletion), *m1404* (−11 bp deletion). **D** The *th*^*m1403*^ genotyping assay reveals a 278 bp fragment in mutant (−/−) and EcoRI-cleaved 167 and 111 bp fragments in WT (+/+). **E** Scheme showing the next in-frame and out-of-frame ATGs after the start ATG in the Th protein. The succeeding in-frame ATG, which could serve as an alternative initiation codon, is at 589 bp (ORF amino acid 196) at the beginning of the Th active aromatic amino acid hydroxylase domain. Translation from this start codon would lead to a hypothetical carboxyterminal protein of about 60% of the Th sequence. AAAH, green: active aromatic amino acid hydroxylase domain. **F** Anti-Tyrosine hydroxylase immunofluorescence in 3 dpf WT siblings and homozygous *th* mutants shows complete absence of Th immunoreactivity in *th*^*m1403/m1403*^ larvae. Dorsal view, anterior at left. Scale bar: 50 µm.

**Fig. 2:**
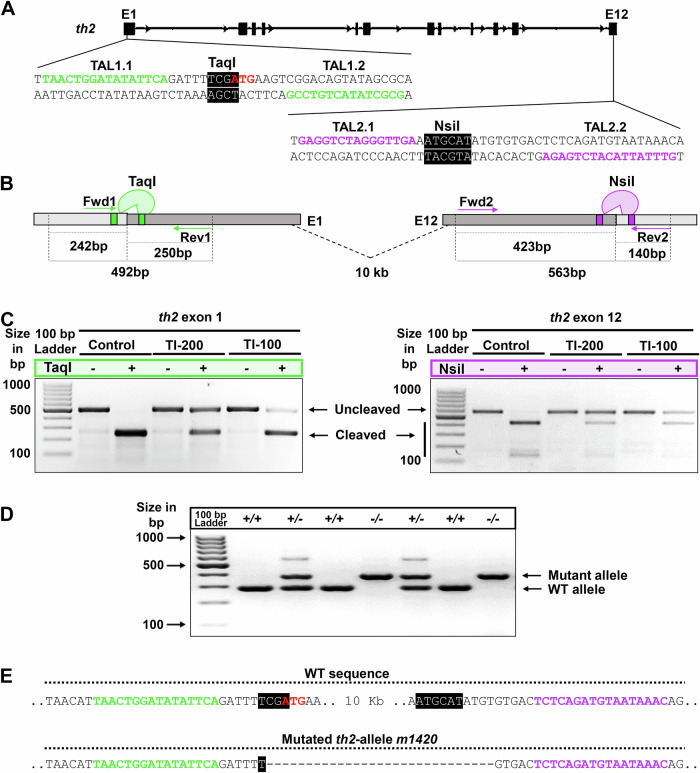
TALEN-mediated strategy for *th2* deletion. **A** TALENs were designed for targeting the *th2-*exon 1 (E1) and *th2*-exon 12 (E12). Black boxes depict the 12 *th2* exons. Close-up of the TALEN-binding regions in exons E1 (TAL1.1 and TAL1.2, green) and E12 (TAL2.1 and TAL2.2, magenta). ATG start codon is shown in red; TaqI (*th2*-E1) and NsiI (*th2*-E12) sites are highlighted. **B** Scheme of restriction fragment length polymorphism (RFLP) strategy for genotyping and expected PCR product sizes; bp: base pairs. (**C**) Digested PCR products amplified from *th2*-E1 and *th2*-E12. Control: non-injected samples; TI-200: TALEN-injected samples with 200 ng/μL; TI-100: TALEN-injected samples with 100 ng/μL; (+) restriction digest; (−) non-restricted; L: 100 bp ladder. **D** Complete deletion of *th2* E1-E12 in the *th2*^*m1420*^ allele. For *th2*^*m1420*^ genotyping, a common reverse primer (5′-ATGCCCCTGGAAATAGGC-3′) and two mismatch forward primers hybridizing either the wild-type (5′-CTTGGGGAGACTGGGACAG-3′) or mutant (5′-CTGTCGCTTGAAGCACCTG-3′) sequences were used, resulting in DNA fragments of 286 bp for wild-type (+/+) and 396 bp for mutant alleles (−/−). **E** DNA sequences from control (WT sequence) and the *th2* deletion (mutated *th2*-allele *m1420*).

To generate a catecholamine-depleted model, we induced loss-of-function mutations in both *tyrosine hydroxylase* paralogous genes, *th* (Fig. 1) and *th2* (Fig. 2). The *th* gene was mutated using a pair of TALENs targeting the beginning of the ORF in exon 1 containing the start codon ATG and a restriction site for EcoRI (Fig. 1A–C). We established a stable mutant line for the allele *th*_*m1403*_ (Fig. 1D), which harbors a 7 bp deletion beginning with the G of the start ATG (A equals bp position 1). In the absence of the native Th ATG start codon, the closest ATG is located out-of-frame at 125 bp, followed by a stop codon after 18 aa (Fig. 1E). Anti-Th immunohistochemistry with a polyclonal zebrafish anti-Th antibody raised against amino acids 92–365^37^ shows the complete absence of Th immunoreactive proteins in *th*^*m1403/m1403*^ larvae (Fig. 1F), demonstrating that the *m1403* allele is a protein Null allele.

To ensure the complete knock-out of *th2*, a deletion of all coding exons was performed (Fig. 2). TALEN-binding regions included a restriction site for TaqI in *th2*-exon 1 and NsiI in *th2*-exon 12 (Fig. 2A–C). The 10 kb deletion allele *th2*^*m1420*^ was confirmed by allele-specific PCRs across the deleted region and sequencing of the exon 1–exon 12 linking fragment (Fig. 2D, E).

To determine whether there could be a maternal rescue by Th or Tyr activity derived from maternal transcription, we analyzed expression data from public databases. For *th* and *th2,* no transcripts have been detected from zygote to 19-somite stage^38^, or in primordial germ cells^39^, excluding maternal transcript rescue. The same sources reveal very low levels of maternal *tyr* transcript in primordial germ cells and the zygote. In addition, low levels of zygotic *tyr* transcription appear to occur even before zygotic genome activation stage^38^, which in mutants would not contribute to Tyr activity. However, by mid-somitogenesis (16–18.5 h post-fertilization), all *tyr* transcripts have been cleared. Therefore, we conclude that there is likely no maternal *tyr* rescue affecting developmental stages analyzed in our study.

**Fig. 3:**
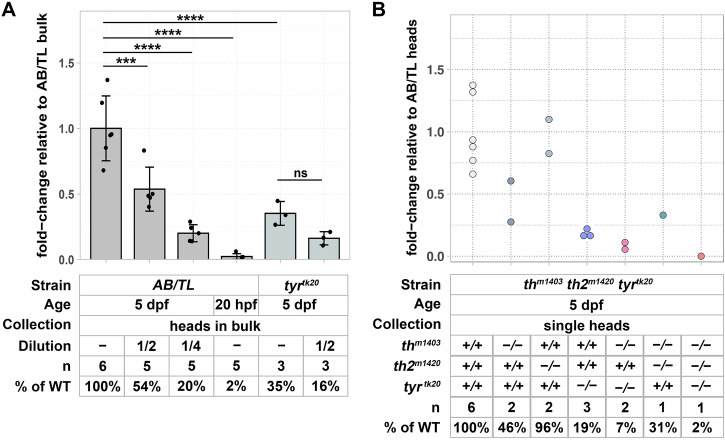
Dopamine depletion in *th, th2, tyr* mutant embryos. **A** Dopamine amount detected by ELISA in 50 heads in bulk. First three bars show sample dilution series of WT *AB/TL* samples and reveal a linear ELISA readout. Measurements were obtained from 500, 250, or 125 µl extracts obtained from 50 heads in bulk of 5 dpf larvae (see “Methods” section). As a negative control, 20 hpf *AB/TL* whole larvae were used, as at this stage Tyr or Th activities are still not expressed. DA concentration was measured from 500 or 250 µl extract from 50 heads prepared in bulk from 5 dpf larvae. Error bars represent the mean and standard deviation. For data and statistical analysis, see Supplementary Fig. 2, Supplementary Tables 1, and Supplementary Data 1–3. **B** Dopamine amounts measured in 50 combined single heads of single, double, and triple mutant *th, th2*, and *tyr* genotypes as indicated. Sample collection was done at 5 dpf using heads pooled after genotyping of trunk material from individual heads (single heads). See Supplementary Data 1–3. **A**, **B** % of WT is based on the mean of all measurements compared to the WT mean (100%). The sample numbers (*n*) in the table give the number of biological replicates for each condition. Statistical analysis in (**A**) was performed using one-way ANOVA (see Supplementary Table 2; n.s. not significant, *p*-value > 0.05; *p-value < 0.05; **p-value < 0.001; ***p-value < 0.0001; ****p-value < 0.00001); Data in (**B**) was not subjected to statistical analysis because the scarcity of the double and triple mutant combinations inhibited the collection of material for additional samples.

We determined the DA content of WT and mutant larval heads using an ELISA assay. A two-step two-fold serial dilution of WT extracts and a two-fold dilution of *tyr* mutant extracts reveals a near-linear relation between the sample amount in the assay and the DA amounts calculated based on ELISA readings (Fig. 3A, Supplementary Tables 1 and 2, Supplementary Data 1 and 2). As a negative control, we used 20 hpf embryo extracts, which should be devoid of DA because *th*, *th2* and *tyr* expression have not started at this developmental stage (Fig. 3A), and indeed did not detect DA above background. ELISA readings of *tyr* mutant heads prepared by this bulk approach revealed a significant reduction of DA in larval heads to about one third of WT levels.

For reference to published DA values, we determined protein content of extracts and calculated pMol DA per mg protein (Supplementary Data 3). WT bulk heads contain 58.1 pMol DA per mg protein (*n* = 3, SD 8.5), while in WT single heads we detected 27.8 pMol DA per mg protein (*n* = 3, SD 7.0), revealing a loss of DA during the more extensive handling involved in single heads as compared to bulked preparation. These values are higher than previously published for zebrafish brain^40^, but lower than values compiled from eight publications on rat striatum^41^, which reported an average of 61.8 (SD 34.4) pMol DA per mg wet brain tissue. However, differences in extraction, detection, and normalization methods may limit comparison of calculated DA content.

To determine relative contributions of each gene to total DA, we performed ELISA on WT and single, double, and triple *th, th2, tyr* mutant combinations. Here, each larva was collected separately, heads frozen and genotypes determined by PCR on tail biopsies. For each genotype, 50 heads (single heads) were then combined into one batch for DA ELISA (Fig. 3B, Supplementary Table 1, Supplementary Data 1 and 2, Supplementary Fig. 2). Due to scarcity of the required mutant genotypes, we were not able to obtain sufficient heads for three independent biological replicates for all genotypes (see “Methods” section), and therefore data in Fig. 3B were not subjected to statistical analysis. The results indicate that in the *th* (about 46% DA of WT; *n* = 2) and *tyr* (19% of WT; *n* = 3) mutants, DA levels are reduced compared to WT. In *th2* mutants (about 96% DA of WT; *n* = 2), only a minor reduction of DA content was observed. *th, th2* double mutants are not sufficient to completely impair DA biosynthesis, as about 31% of WT DA levels (*n* = 1) remained in the double mutants. In contrast, *th, tyr* double mutants show a much stronger loss of DA, with only about 7% of WT (*n* = 2) remaining. *th, th2, tyr* triple mutants (*n* = 1) reveal absence of DA, as the measured DA amount is not different from 20 hpf WT negative control samples (Fig. 3A, B). Since DA is the first CA synthesized from L-DOPA, absence of DA indicates that triple mutants are also devoid of NA and adrenaline.

The data surprisingly reveal that *tyr* has the largest relative contribution to total head DA, *th* accounts for most of the remaining DA, while *th2* at 5 dpf likely contributes only a few percent of total DA, consistent with the relatively small number of *th2-*expressing cells at 5 dpf. The abundance of *th* mRNA is about 8-fold higher than for *th2* in 5 dpf larvae (https://www.ebi.ac.uk/gxa/experiments/E-ERAD-475)^38^, similar to the 10-fold difference in contribution to total DA amount we observe.

### Catecholaminergic cluster development in *th th2 tyr* triple mutant larvae

**Fig. 4:**
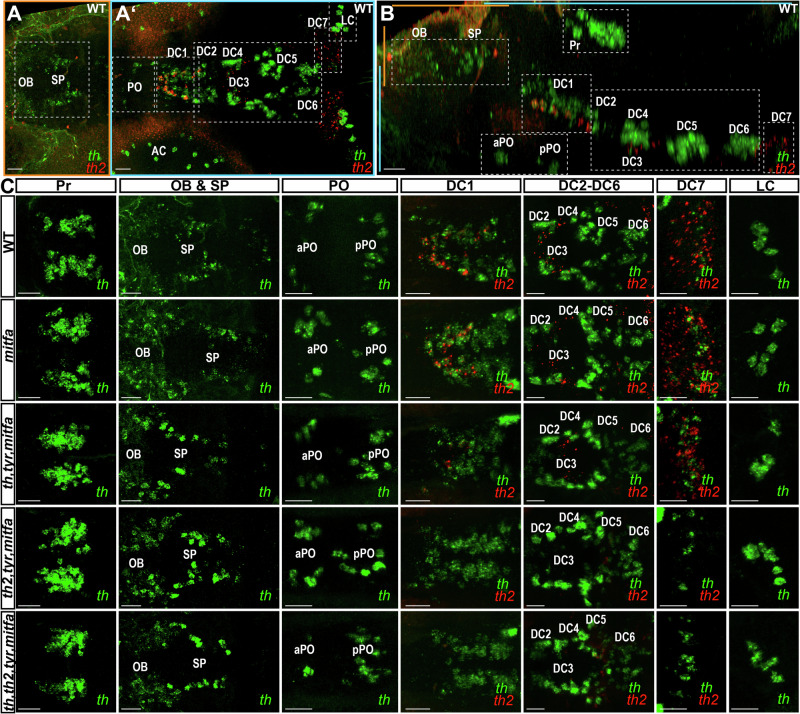
Development of CA neurons in *tyr, th* and *tyr, th2* as well as triple mutant larvae. **A**, **B*** th* (green) and *th2* (red) expression patterns in 5 dpf WT whole-mount larvae. mRNA detected by HCR and recorded as dorsal view (horizontal) confocal stacks (**A**, **A**′) and sagittal view prepared from the orthogonally resliced stack (**B**). Planes used for (**A**) and (**A**′) do not include the dorsalmost pretectal DA group depicted in (**B**). Images show maximum intensity projection; dimensions of projection in (**A**) are indicated by orange bars in (**B**), and of (**A**′) by blue bars. **C** Maximum intensity Z-projections of confocal substacks for specific CA clusters with x, y, and z dimensions indicated by dashed boxes in (**A**), (**A**′), and (**B**). Genotypes are indicated in boxes. Mitfa mutants lack neural crest-derived melanocytes and are used as a second reference genotype next to wild-type AB/TL larvae. *th2* expression cannot be detected in mutant combinations including *th2−/−* because the transcribed region is deleted in the mutant chromosomes. In (**C**), image brightness was adjusted for each cluster, but similar for all genotypes. Scale bars 20 µM for all panels. *n* = 2 for WT, *n* = 3 for all other genotypes. For cell counts and statistical analysis, see Supplementary Fig. 3, Supplementary Tables 3 and 4, Supplementary Data 4 and 5. AC amacrine cell, DC1-7 diencephalic groups 1–7, LC locus coeruleus, OB olfactory bulb, PO preoptic region (including anterior (a) and posterior (p) PO group), Pr pretectum, SP subpallium.

To investigate a potential impact of absence of CA activity on DA neuron development, we analyzed *th* and *th2* mRNA expression by whole-mount fluorescent in situ hybridization (WISH, by HCR-RNA-FISH; Fig. 4, Supplementary Fig. 3 and Supplementary Tables 3 and 4, Supplementary Data 4 and 5). Since the *th*^*m1403*^ allele impairs Th translation but not *th* transcription, *th* WISH labels DA neurons in *th th2 tyr* triple mutant larvae. However, in the *th2*^*m1420*^ allele, the *th2* transcribed region is deleted, and therefore transcripts can only be detected in embryos not mutant for this allele. We also wanted to determine whether there is a potential influence of melanocyte-derived L-DOPA, and therefore bred into the triple mutant line *mitfa*^*s184*^ that is devoid of melanocytes but develops a pigmented epithelium of the retina^42^. We did not detect statistically significant differences in CA neuron counts between WT and neural crest-derived melanocyte-deficient *mitfa* mutant larvae, except for clusters DC3 and OB. However, comparison of *th* expression in WT and *mitfa, th, th2, tyr* combined mutants did not reveal significant differences, arguing that there are in fact no biologically significant differences in CA neuron development between WT and *mitfa*^*s184*^ mutants. Further, differences in *th* cell count observed between WT and *mitfa, tyr, th* and/or *th2* mutant larvae (DC3, DC4, DC6 clusters) were not observed when comparing to *mitfa* mutants. A non-significant tendency for reduced *th* cell counts in *th* mutant combinations (DC3, DC4, LC, PO, SP cluster) may also be caused by *th*^*m1403*^ transcripts being potentially less stable due to nonsense-mediated mRNA decay^43,44^, resulting in some *th-*expressing cells of clusters with low expression levels escaping detection. We conclude that there are no biologically significant differences in CA cell numbers due to loss of CA activity.

**Fig. 5:**
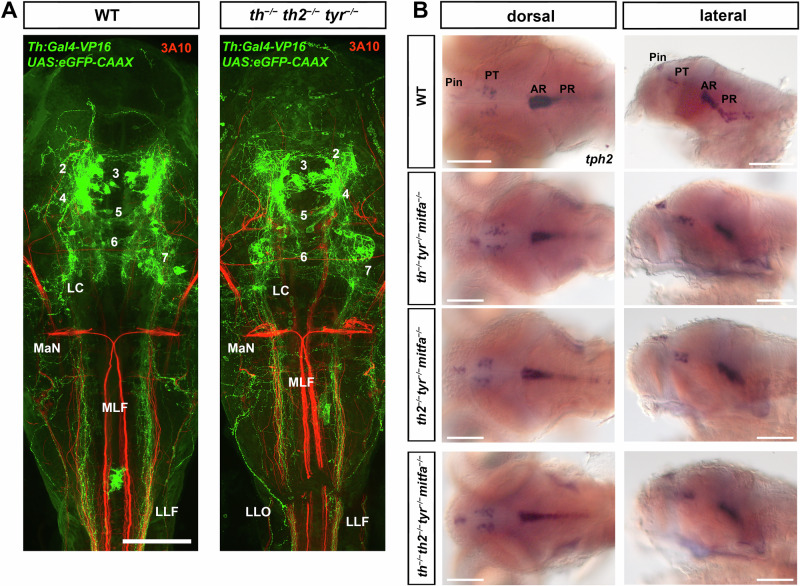
Development of CA projections and serotonergic cell clusters in CA-deficient larvae. **A** Anti-GFP (green) and anti-3A10 (red) immunofluorescence staining in 3 dpf *Tg(th:Gal4-VP16)*^*m1233*^*, Tg(UAS:eGFP-CAAX)*^*m1230*^ transgenic larvae show CA tracts and axonal projections (green) as well as other axonal scaffolds (red). Genotypes as indicated (*n* = 2 for each genotype), dorsal view, anterior up. Scale bar: 100 µm. **B** Whole-mount in situ hybridization showing *tph2* expression in 5 dpf larvae of genotypes as indicated: WT (*AB/TL*), and the following mutant combinations in *mitfa−/−* background: *tyr* double mutants, *th2 tyr double* mutants, and *th th2 tyr* triple mutants. Dorsal and lateral views as indicated. *N* = 2 for *th tyr mitfa* and *n* = 3 for all other genotypes. Bleaching of pigment cells was performed post-fixation for WT embryos. Scale bar: 100 μm. 1–7 ventral forebrain DA groups 1–7, LC locus coeruleus, LLF lateral longitudinal fascicle, LLO lateral line organ, MaN Mauthner neuron, MLF medial longitudinal fascicle, MO medulla oblongata, Pin pineal stalk, PT pretectum, AR anterior raphe, PR posterior raphe.

To determine whether CA deficiency affects axonogenesis of CA neurons, we crossed a 3 dpf transgenic line expressing membrane-anchored GFP in *th* cells (*Tg(th:Gal4-VP16)*^*m1233*^, *Tg(UAS:eGFP-CAAX)*^*m1230*^) into a *th, th2, tyr* heterozygous line. We combined anti-GFP immunofluorescence for CA axons with anti-neurofilament 3A10 stain for the general axonal scaffold. Staining of axonal projections shows no major differences between WT and CA-deficient larvae (Fig. 5A). In triple mutants, axons of the medial longitudinal catecholaminergic tracts project caudally from the posterior tuberculum through midbrain and hindbrain just medial to the lateral longitudinal fascicle into the spinal cord. The lateral line and other peripheral sense organs also show normal innervation from catecholaminergic tracts^45^. Thus, CA axonogenesis and target region innervation do not depend on CA activity. However, we note that second transmitter systems in CA neurons may support potential activity-based mechanisms in target innervation.

### Raphe serotonergic neurons and spinal motor neuron numbers are not affected by catecholamine deficiency

Raphe serotonergic neurons receive DA input from the ventral tegmental area in mice^46,47^, and show extensive crossregulation with DA systems^48^. In zebrafish, quinpirole D2/D3 DA receptor activation leads to a decrease in serotonin^49^. Therefore, we investigated whether there is a change in tryptophan hydroxylase encoding mRNA (*tph2)* expression that controls serotonin biosynthesis. However, we find that expression of *tph2* appears normal in anterior and posterior raphe, pretectum, and pineal stalk in *th, th2, tyr* triple mutants (Fig. 5B).

**Fig. 6:**
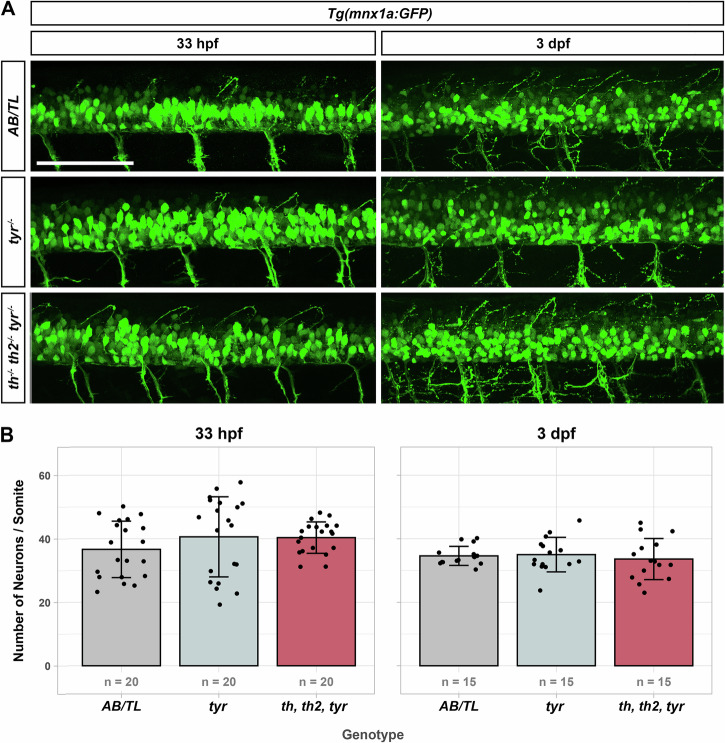
Primary and secondary motor neuron numbers are not affected by catecholamine depletion. **A** GFP immunofluorescence in *Tg(mnx1a:GFP)*^*ml2Tg*^ embryos shows no difference between the genotypes examined, neither at 33 hpf nor at 3 dpf. Lateral view, anterior at left, dorsal up. Scale bar 100 µm. **B** *mnx1a: GFP-positive* primary (PMN) and secondary (SMN) motor neurons were counted at 33 hpf in 4 somites (7, 8, 9, and 10) of 5 embryos each, and at 3 dpf in 3 somites (7, 8, and 9) of 5 embryos each (Supplementary Tables 5 and 6). Given that PMN and SMN form independently in each somite, we considered each somite count as a biological replicate. At 3 dpf *Tg(mnx1a:GFP*) expression is low in some cells, which were not counted, resulting in smaller cell numbers counted at 3 dpf. For PMN: *F* Welch (2, 33.3) = 1.37, *p* = 0.27. For SMN: *F* Welch (2, 24.99) = 0.21, *p* = 0.81 (Supplementary Tables 5 and 6). Means with SD are plotted. Comparison of cell counts per larva also reveals no significant differences (33 hpf: *n* = 5, ANOVA *p* = 0,745; 3 dpf: *n* = 5, *p* = 0.864).

DC2 and DC4 A11-type DA neurons with descending diencephalospinal projections have been reported to promote motor neuron (MN) generation in the zebrafish spinal cord^50^. These experiments used Morpholino knockdown of *th* and DA signaling pathway components, as well as pharmacological DA antagonists, and therefore DA was not completely eliminated. We tested whether complete elimination of DA affects spinal MN development. We crossed *Tg(mnx1:GFP)*^*ml2Tg*^, which labels primary and secondary MNs, into the *th, th2, tyr* line, performed anti-GFP immunofluorescence on embryos fixed at 33 hpf or 3 dpf (Fig. 6A), and counted labeled MNs in spinal sectors correlating in length to one somite. *mnx1:GFP* brightly labels new primary and secondary MNs, but expression decreases in older MNs, such that MN counts were lower at 3 dpf. The relative immunofluorescence intensities were not different between mutants and WT. No significant differences were detected when counts of MNs in WT, *tyr* and triple mutant genotypes were compared, neither in the number nor location of MNs (Fig. 6B, Supplementary Tables 5 and 6). Therefore, we did not observe an effect of DA deficiency on MN development, suggesting that the previously reported requirement for DA^50^ may actually have been caused by experimental interference with other non-DA regulatory systems. Alternatively, genetic compensation may mask a phenotype in mutants but not morphants^51^. Serotonin also controls motor neuron development^52^, and may thus indirectly contribute to compensation.

### Catecholamines are necessary for hatching gland cell granule secretion

**Fig. 7:**
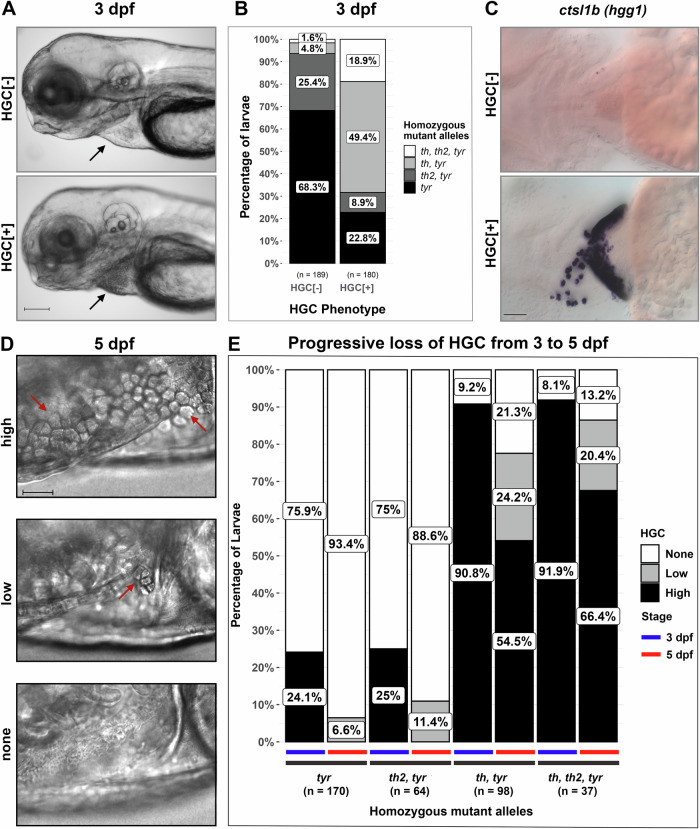
HGC retention is a characteristic phenotype of *th* mutant larvae. **A** HGC phenotype at 3 dpf larvae obtained from a *th*+*/-, th2* + */-, tyr−/−* cross, live images, lateral view, anterior at left. HGC[-]: absence of granule-containing cells in *th +/*−*, th2 +/+, tyr−/−* larva; HGC[+]: retention of granule-containing cells in triple mutant larva. Black arrow points toward the pericardial region where HGCs are located. Scale bar 100 μm. **B** Percentages of larval *th* and *th2* genotypes found in HGC phenotypes of *tyr*−/− larvae (for *tyr* mutants, *th* and/or *th2* may be heterozygous; for *th, tyr* mutants, *th2* may be heterozygous; for *th2*, *tyr* mutants, *th* may be heterozygous). The HGC-retention phenotype correlates with the *th* genotype (Pearson’s chi-square χ^2^ (3) = 152.7, *p*-value < 6.88e-33, *V* Cramer = 0.64, CI 95% [0.55, 1.00]). For data, see Supplementary Table 7. **C** WISH showing *ctslb* (*cathepsin Lb;* previously *hgg1*) expression in larvae with either HGC[−] in *th*+*/+, th2*+*/+, tyr−/−* larvae or HGC[+] in triple mutant larvae at 4 dpf. Scale bar 40 μm. **D** Live images of the hatching gland region in 5 dpf larvae from a *th*+*/−, th2*+*/−, tyr−/−* cross (anterior at left, dorsal up). Larvae classified as HGC[+] at 3 dpf, when reanalyzed at 5 dpf, reveal progressive HGC loss during larval development. Red arrows point to exemplary HGCs. High, low, and none describe the different degrees of HGC retention observed. Genotypes shown are “High” HGC retention: *th−/−, th2*+*/−, tyr−/−*. “Low”: *th*+*/+, th2*+*/+, tyr−/−*. “None”: *th*+*/*−*, th2*+*/*−*, tyr−/−*. Scale bar 20 μm. **E** HGC retention in larvae obtained from a *th*+*/*−*, th2*+*/*−*, tyr−/−* cross was analyzed at 3 dpf and again at 5 dpf. The graph shows the percentage of larvae classified as high, low, or no HGC retention for each genotype at 3 dpf (bars marked by blue line) and 5 dpf (bars marked by red line); for genotypes see explanation in (**B**). For data, see Supplementary Table 8.

At a gross anatomical level, CA-free larvae appear to develop largely normally up to 5 dpf (Fig. 5B). However, a careful inspection revealed major differences in hatching gland cell (HGC) retention during larval stages (Fig. 7A). The secretory hatching gland epithelium produces and releases proteolytic enzymes involved in the breakdown of the chorion during hatching^53^, and HGCs disappear following this process. Therefore, most HGCs are lost in larvae older than 2 dpf: 200 WT larvae analyzed at 3 dpf were all completely devoid of HGCs. In contrast, in *tyr−/−* larvae we found 9.5% HGC retention at 3 dpf (*n* = 16 out of 168), and 2.9% HGC retention at 5 dpf (*n* = 5 out of 168). Thus, in *tyr−/−* mutants, a small but significant portion of larvae retains HGCs to 5 dpf. To efficiently recover triple mutants for our analysis, we used a *th*+/−*, th2*+*/−, tyr−/−* incross, with all progeny *tyr* mutant. We observed that 24.7% of the progeny from this cross (*n* = 39/158) had HGCs still detectable at 3 dpf (HGC[+], Fig. 7A). Genotyping revealed a correlation between HGC-retention phenotype and *th* genotype (Fig. 7B, Supplementary Table 7). 68.3% of the HGC[+] larvae were *th* mutants (*th, tyr* double or *th, th2, tyr* triple mutants), while only 6.4% of all HGC[-] larvae were *th* mutants. Accordingly, 93.6% of the HGC[-] larvae had at least one WT *th* allele (Fig. 7B). These results show that animals lacking Th have impaired mechanisms of HGC granule secretion, demonstrating that *th* gene activity is necessary for the normal function of HGCs. We analyzed expression of the HGC specific marker *cathepsin Lb* (*ctslb)*^54^ and found expression maintained in HGC[+] larvae at 4 dpf (Fig. 7C). When we reexamined 3 dpf HGC[+] larvae at 5 dpf (Fig. 7D), we observed three different phenotypes in these larvae: retention of all HGCs (High), few HGCs (Low) and complete absence of HGCs (None, Fig. 7D), revealing progressive loss of HGCs during larval stages in some genotypes (Fig. 7E). After genotyping all larvae, we noticed that high retention at 5 dpf was only observed in *th−/−* mutants (Fig. 7E, Supplementary Table 8). However, a fraction of *tyr* single mutant larvae also shows low HGC retention. Tyrosinase-derived L-DOPA more likely contributes to tissue-level interstitial DA, rather than affecting synaptic transmission. We hypothesize therefore that tissue CA levels may directly affect HGC secretion, potentially independent of the established DA-prolactin neurosecretory hypothalamic-hypophyseal axis^53,55,56^. To elucidate whether DA or NA receptors on HGCs may be able to mediate direct effects of systemic CA levels, we queried the Daniocell database (www.daniocell.nichd.nih.gov)^57^ for expression of CA receptors in *ctsl1b* expressing HGC clusters (clusters axia.15, axia.18, axia.20; Supplementary Fig. 4A). The results indicate that HGCs express *drd5a* and *adrb3a* at higher levels, as well as *adrb1, adrb2a* at lower levels, which are all adenylate cyclase activating receptors (Supplementary Fig. 4B–G). Thus, DA or NA may potentially stimulate HGC secretion directly.

### Catecholamines regulate heart rate and heart rate variability upon thermal challenge

**Fig. 8:**
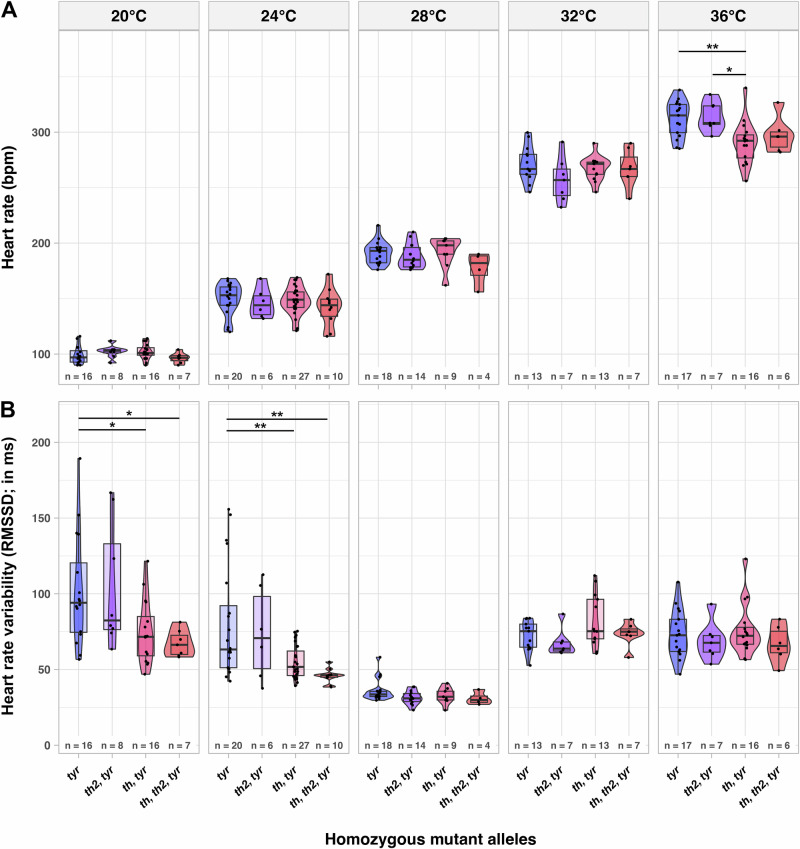
Reduced catecholamine levels affect normal HR and HRV adaptations during a thermal challenge. **A** Heart rate (HR) and **B** heart rate variability (HRV) for *th* and *th2* genotypes in a *tyr* mutant background (3 dpf) at different temperatures. For data, see Supplementary Table 9. Statistical analysis was performed using two-way ANOVA type III with Tukey’s multiple-pairwise comparisons test (see Supplementary Data 6 and 7, and Supplementary Table 10). Only significant differences are shown (ns: *p*-value > 0.05; *p-value < 0.05; **p-value < 0.001). Boxes represent the interquartile range (25th–75th percentiles, 50% of the data), the central line corresponds to the median, and the vertical lines show the spread of the remaining data.

In response to acute physiological stress, the mammalian sympathetic nervous system triggers a multisystemic CA reaction responsible for the physiological changes needed to cope with sudden environmental threats^58^. Sympathetic modulation of heart performance involving CAs is key for a fast fight-or-flight response. Since our model is completely devoid of CAs, we investigated how CA deficiency affects basic cardiac parameters, such as heart rate (HR) and heart rate variability (HRV), during acute variation of temperature conditions^59,60^. Heartbeat was recorded in 3 dpf non-anaesthetized and freely moving larvae at control temperature (28 °C) and at four thermal challenges (20 °C, 24 °C, 32 °C, and 36 °C), each measured 1 min after sudden temperature shift (Supplementary Table 9). At 28 °C, we observed in *tyr−/−* larvae an HR of 191 beats per minute (bpm; *n* = 18, STDEV 9.9 bpm), similar to previous reports for WT larvae at this developmental stage^61^. HRV was 35.95 ms (*n* = 18, STDEV 7.14 ms), also similar to previously reported values (30 ms)^62^. HR and HRV are not significantly different in *AB/TL* WT compared to *tyr−/−* larvae (Supplementary Fig. 5). As expected from an ectotherm animal, we observed a positive correlation between temperature and HR (Supplementary Fig. 6A). Considering all temperature conditions and all genotypes, two-way ANOVA revealed a significant interaction between temperature and genotype for HR and HRV (HR *p* = 0.00386, HRV *p* = 0.00078; Supplementary Data 6 and 7, Supplementary Fig. 6B), indicating that both HR and HRV responses to temperature changes depend on the genotype of the fish. To specifically identify which genotypes contribute to these differences, Tukey’s test was conducted and revealed that *th*, *th2*, *tyr* triple mutants exhibited significant differences in HR and HRV when compared to *tyr* single mutants across specific temperature conditions (Tukey’s multiple-pairwise comparisons, HR *p* = 0.0259, HRV *p* = 0.00011, Supplementary Table 10). When analyzing specific temperatures, no statistically significant differences in HR or HRV were detected when mutant genotypes were compared at standard breeding temperature (28 °C) (Fig. 8A, B and Supplementary Data 6 and 7 D terms 17–206 rows highlighted yellow), while a trend for reduced HR can be observed for triple mutants. However, for HR, at 36 °C *th, tyr* double mutants show a significantly lower HR than *tyr* mutant or *th2, tyr* mutant siblings (Supplementary Data 6 rows highlighted green). For HRV, both *th, tyr* double and *th, th2, tyr* triple mutants have a significantly smaller HRV at 24 °C and 20 °C when compared to *tyr* single mutants (Fig. 8B, Supplementary Data 7 rows highlighted blue). Our data thus reveal that in zebrafish larvae, CAs contribute to temperature-dependent HR and HRV modulation.

### Catecholamines modulate spontaneous and stimulus-triggered movement

DA plays a crucial role in zebrafish normal locomotor development^63,64^ as well as in sensory processing^65^. Therefore, we assessed if CA depletion specifically affects locomotor activity. To avoid effects of genetic heterogeneity in the non-inbred zebrafish strains, in each experiment we used larvae from a single *th*+*/−*, *th2*+*/−, tyr−/−* cross. To recover sufficient numbers of triple mutant embryos from an individual cross, we used *tyr−/−* larvae as internal reference instead of WT larvae. *tyr* mutant and WT *AB/TL* larvae have similar spontaneous motor activity based on analysis of distance traveled (Supplementary Fig. 7, Supplementary Table 11).

**Fig. 9:**
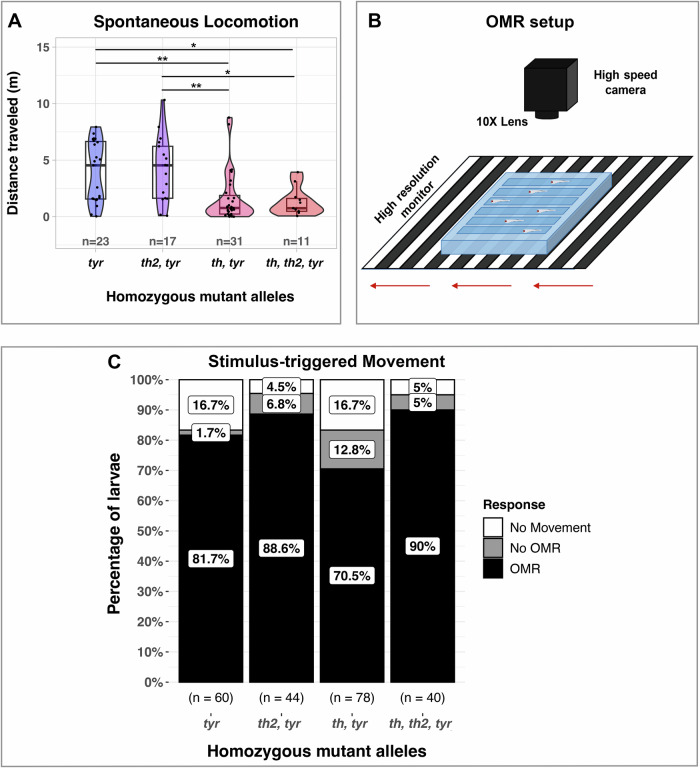
th-derived catecholaminergic activity is required for normal locomotor activity. **A** Spontaneous locomotor activity measured as distance traveled in 1 h is reduced in 5 dpf *th* mutant compared to *th2* mutant or *tyr* control larvae (Supplementary Tables 12; see also Supplementary Fig. 5 and Supplementary Table 11). Statistical analysis was performed using a Kruskal–Wallis rank sum test (χ^2^ (3) = 19.95, *p* = 0.000174, effect size (ε^2^ (rank) = 0.25, CI 95% [0.12, 1.00]), with Dunn’s pairwise comparisons test. *: Only significant differences are shown (ns: *p*-value > 0.05; *p-value < 0.05; **p-value < 0.001). Boxes represent the interquartile range (25th–75th percentiles, 50% of the data), the central line corresponds to the median, and the vertical lines show the spread of the remaining data. **B** Schematic representation of optomotor response (OMR) setup. Red arrows indicate grating movement. **C** Comparison of response types reveals that all genotypes perform OMR with minor differences in efficiency (Pearson’s chi-square χ^2^ (6) = 14.117, *p*-value < 0.028, *V* Cramer = 0.14, CI 95% [0.00, 1.00]). See Supplementary Table 13.

We first determined spontaneous movement measured as distance swam in 1 h by each larva in a bright environment without specific stimuli. We observed that mutant combinations including *th* (*th−/−, tyr−/−* and *th−/−, th2−/−, tyr−/−*) show a significantly reduced spontaneous locomotion compared to *tyr* siblings (Fig. 9A, Supplementary Table 12). In contrast, *th2 tyr* mutants showed no significant locomotor activity differences when compared with *tyr* siblings (Fig. 9A). These results indicate that CA modulation of spontaneous swimming depends predominantly on Th but not on Th2 activity. Next, we examined whether a visual stimulus-triggered response was also affected by CA depletion. We analyzed the optomotor response (OMR), a reflexive behavior where the animal follows the direction of whole-field motion^66^. Moving gratings were presented to individual larvae from below, orthogonal to the longitudinal swim rails, and larvae were recorded (Fig. 9B). We classified the motor outputs in three groups: OMR (moving in the direction of the stripes), no-OMR (moving without engaging in OMR), and no-movement (remaining still during the entire trial; Fig. 9C, Supplementary Table 13). For all genotypes, the vast majority of larvae engage in OMR. However, we observed small differences in the percentages of animals that do not engage in OMR or do not move. Surprisingly, for all genotypes that contain *th2−/−* mutants, larvae appear to have slightly higher OMR response percentages, which may relate to *th2* being mostly expressed in the hypothalamus, where basic behavioral states including sleep and alertness are regulated. However, the relatively small differences may not justify a mechanistic interpretation.

**Fig. 10:**
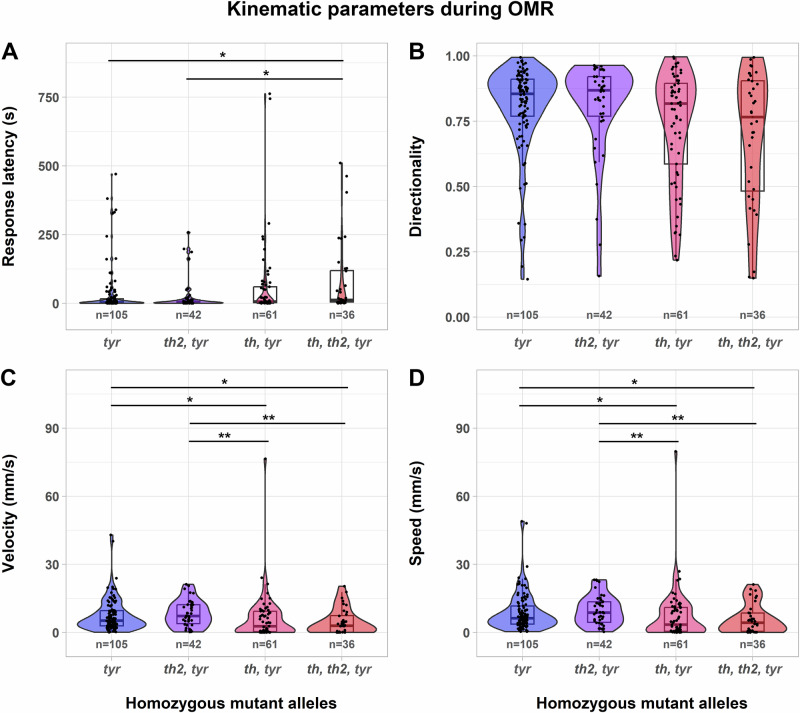
Catecholamine deficiency affects OMR kinematic parameters. Kinematic parameters during optomotor response (OMR) for each larval genotype at 5 dpf. See also Supplementary Data 8, Supplementary Table 14, and Supplementary Fig. 8. **A** Latency in OMR initiation: Delay in responding to the stimulus onset (χ^2^ Kruskal–Wallis (3) = 14.02, *p* = 0.00288, effect size (ε^2^) = 0.06, CI 95% [0.03, 1.00]). **B** Directionality: Quotient between displacement and trajectory (χ^2^ Kruskal–Wallis (3) = 7.73, *p* = 0.05, effect size (ε^2^) = 0.03, CI 95% [0.01, 1.00]). **C** Velocity (vector): rate of change in larval position from beginning of movement to end of track (χ^2^ Kruskal–Wallis (3) = 18.26, *p* = 0.00039, effect size (ε^2^) = 0.08, CI 95% [0.03, 1.00]). **D** Speed (scalar): total trajectory over time moved (χ^2^ Kruskal–Wallis (3) = 17.50, *p* = 0.000557, effect size (ε^2^) = 0.07, CI 95% [0.04, 1.00]). All parameters were calculated considering the position the larvae had at the moment of OMR initiation until reaching the end of the plate. Statistical analyses were performed using Kruskal–Wallis with Dunn’s pairwise comparisons and the Holm *p*-adjustment method (Supplementary Table 14): ns: *p*-value > 0.05; *p-value < 0.05; **p-value < 0.001. Boxes represent the interquartile range (25th–75th percentiles, 50% of the data), the central line corresponds to the median, and the vertical lines show the spread of the remaining data.

Since OMR depends on both visual and motor systems function, DA impairment may differentially affect the kinematic parameters composing the OMR. We measured the trajectory and displacement as well as the time taken by the larvae to reach the end of the OMR track. The latency in responding to the stimulus onset was also determined (Supplementary Data 8 and Supplementary Table 14). *th, th2, tyr* mutants have a higher latency in initiating an optic-flow-directed movement than *tyr* or *th2, tyr* mutant siblings (Fig. 10A). These data reveal that OMR latency is increased by the lack of Th and Th2 activity. Based on trajectory, displacement, and time taken, we calculated directionality (Fig. 10B), velocity (Fig. 10C), and speed (Fig. 10D). Both velocity and speed are significantly reduced in *th* mutants (*th, tyr* and *th, th2, tyr*) when compared with the *tyr* control or *th2, tyr* mutant siblings. Upon excluding outliers, directionality also appears significantly reduced (Supplementary Fig. 8). Lack of *th2* activity does not affect any of the four parameters significantly. While latency and directionality are related to proper detection and processing of visual cues, the speed and velocity largely depend on downstream motor pathways, suggesting that CA modulation affects both visual and motor components of the OMR.

## Discussion

We developed a CA-deficient zebrafish model by combining mutations in all genes involved in L-DOPA synthesis, *th, th2,* and *tyr*. Surprisingly, our data indicate that Tyr activity contributes L-DOPA to more than half of total DA synthesis in larvae. Th activity also contributes nearly half of total DA, while Th2 contributes only between 5% (compared to WT) and 15% (in *th* mutants). The strong contribution of *th* correlates with the broader expression of *th* compared to *th2*^30–32^, and with higher total *th* mRNA expression^30^. Previous *th* knock-down experiments reported smaller contributions of *th*^67^, which may have been caused by partial Morpholino knockdown. Comparison of DA in *th* and *th2* knock-down experiments also supported a stronger contribution of *th* to total DA at 5 dpf^68^. The relatively strong contribution of Tyr activity in zebrafish larvae is in contrast to data from mice, which showed that in TH-null mice only 2-3% of WT DA levels are detected in the brain, and analysis of TH-null TYR-null albino mice revealed that 80% of this residual DA originates from TYR activity^13^. The observed difference in Tyr contribution to total DA may at least in part be explained by the fact that we report measurements from whole heads that include skin melanocytes and pigmented epithelium of the retina, while the measurements in mice referred to dissected brains. L-DOPA extracted from pigment cells may have been in part converted by DDC to DA during the isolation procedure. However, the measurements in *tyr th* and *tyr th th2* mutants reveal the relative contributions of Th and Th2 to total DA in the absence of Tyr.

Our analyses so far reveal no major anatomical differences between WT and *th, th2, tyr* triple mutant larval nervous system development at 5 dpf. Importantly, it appears that the full complement of catecholaminergic neurons develops. CA depletion also does not appear to affect global brain axonal scaffold formation, and DA neurons develop anatomically normal projections, including the long diencephalospinal DA tracts and peripheral projections to sense organs. Similarly, an anatomically normal midbrain dopaminergic system has been reported for DA-deficient mice^8^, while mutant embryos die during mid-gestation, likely due to cardiovascular failure. Synaptic activity plays a pivotal role in neuronal survival by coupling neurotransmitter release with neurotrophin uptake^69^. We infer that, despite lacking CAs, release of second transmitters such as glutamate or GABA^17^ may support survival, axonal projections, and potentially synapse maintenance in zebrafish CA neurons. Thus, our model enables future analyses of the impact of lack of CA neuromodulation on specific neural circuits, but, importantly, also insights into non-CA activities of CA neurons, given that selective CA depletion may not eliminate other functions mediated by GABA or glutamate second transmitter systems in these CA neurons.

DA has been previously suggested to be required for proper development of zebrafish primary and secondary spinal motor neurons^50^. However, we find no differences in spinal motor neuron numbers in CA-deficient larvae, indicating that DA is not necessary for motor neuron development at larval stages. The postulated effects of DA on motoneuron development were suggested to be triggered by DA binding to D4a, a D2-like receptor, reducing cAMP levels and PKA activity, a negative regulator of Hedgehog signaling, and thus activating Hedgehog signaling that stimulates motor neuron development^70,71^. Considering that cAMP and PKA are regulated by several hormones and neurotransmitters^72^, we propose that functional redundancy of GPCR and metabolic signaling networks may compensate for loss of DA in our genetic model, resulting in normal motor neuron development. Such a compensatory mechanism may involve serotonin, which also has been shown to promote motor neuron development in zebrafish^52^. We find that the *tph2* expression pattern is not affected in triple mutants, in line with previous findings on *tph1* being unaffected by *th2* knockdown^68^, indicating normal formation of serotonergic neurons.

CAs have also previously been implicated in mammalian stem cell systems, including sympathetic nervous system CA signaling in hematopoietic stem cell specification^73^. However, previous work already showed that in zebrafish earlier neural crest-derived signals rather than sympathetic CA may induce hematopoietic stem cell specification^74^. Therefore, while CAs may contribute to defined cell specification events, in the absence of CAs this requirement may be compensated by parallel signaling pathway activities.

Another physiological parameter affected by CA depletion is HGC release. According to Schoots et al. (1983), dopamine indirectly inhibits hatching enzyme secretion by controlling prolactin release through binding to D2-like receptors on lactotrophic cells^55,56^. However, our finding of HGC retention in CA-deficient larvae indicates additional levels of CA-dependent direct HGC regulation. Even though HGCs do not express D2-like family receptors, they do express high levels of *drd5a* and *adrb3a*, as well as lower levels of *adrb1* and *adrb2a*^57^. Both D1-like family receptors and β-adrenergic receptors are stimulatory G protein-coupled receptors (GPCRs), which activate the cAMP/PKA signaling cascade regulating secretion^75^. Thus, our results suggest the presence of a parallel pathway with direct and positive control of CAs on HGCs, using potentially both DA and NA. A direct action of CAs on HGC secretion is also supported by our observation that *tyr* single mutants show enhanced HGC retention, with Tyr more likely acting through CA tissue levels rather than the hypothalamic-pituitary axis. We note that with respect to temporal control of development, melanocyte differentiation and Tyr expression correlate with HGC release in larval development. Thus, HGC release may not, or not exclusively, be regulated by neuronal CAs.

Similar to mammals, zebrafish cardiovascular function is regulated by CAs and highly sensitive to adrenergic drugs^76^. Since zebrafish are ectothermal, metabolic and physiological processes depend directly on environmental temperature^58^. HR increases in direct relation with temperature^60^, which we also observed for all mutant genotypes. Despite zebrafish being ectothermal, their sinoatrial nodes have structurally and molecularly conserved features compared to mammals^77^, whose heartbeat is generated by autorhythmic cardiomyocytes of the sinoatrial node, which do not depend on CAs. When we analyzed HR in individual CA-depleted genotypes across all temperatures, we observed a small but significant HR reduction, which at a specific temperature appears significant only at 36 °C. This may reflect a requirement for activation of sympathetic NAergic components, as the effect we observe is similar in strength to HR reductions reported for *adrb1* adrenergic beta receptor mutants^61^.

Regarding HRV, we observed that compared to standard temperature (28 °C), both temperature decrease and increase cause an increment in HRV. Surprisingly, *th* mutants at low temperature show lower HRV and thus higher rhythmicity compared to *tyr* control siblings. Reduced HRV in humans is usually linked to cardiovascular disease and is used as a clinical marker to assess autonomic dysregulations. A dynamic balance of vagal and sympathetic input controls HRV, predominantly modulated by cholinergic and noradrenergic activities^78^. Given that the noradrenergic component is depleted in our model, our results potentially reflect the reduced adaptability of CA mutants to changes in environmental conditions.

DA is also an important modulator of central sensory and motor systems. As in mammals, zebrafish DA systems modulate behavioral responses, including locomotor development^63^ and sensory processing^65^. In concordance with these findings, our results showed that loss of Th activity elicits a reduction in spontaneous swimming. The basic commands for executing rhythmic locomotor outputs rely on spinal central pattern generator activity^79^, which is subject to refinement by supraspinal modulatory inputs, including DA^80^. In zebrafish, the diencephalospinal DA tracts^81,82^ project exclusively from posterior tubercular DC2 and DC4 DA neurons, which solely express *th* but not *th2*, and correspond to the mammalian A11 DA system. Our results are concordant with experiments carried out on 5 dpf larvae, where laser ablation of DC2 and DC4/5 groups elicited a reduction in swimming behavior^83^.

Interestingly, in larvae lacking Th2 activity, we did not observe an effect on larval locomotion. In contrast, McPherson et al.^64^ reported that chemogenetic ablation and optogenetic activation of *th2* neurons at 8 dpf affect swim bout initiation frequency. This apparent incongruence with our results could be explained by overlapping expression of *th* and *th2* in the DC7 DA group and in *th2* neurons of the posterior recess^31^. Dopamine transporter may mediate uptake of DC7 *th-*derived DA into posterior recess *th2* neurons in *th2* mutants, potentially rescuing some activities of *th2* DA neurons. Therefore, our experiments may not allow us to define *th-* or *th2-*specific phenotypes, but rather reflect phenotypes caused by a decrease in total interstitial DA levels in the brain. Further, chemogenetic ablation or optogenetic activation of posterior recess *th2* neurons^64^ abolishes both DA and second transmitter, likely GABAergic, activity of these neurons, while in *th2* mutant larvae we expect second transmitter systems in these neurons to still be active.

Upon visual stimulation with moving gratings, *th* mutants showed an abnormal OMR, including reduced directionality, speed, and velocity, while latency in response was considerably increased. Since the execution of optic-flow oriented movement requires the engagement of both sensory and motor pathways, lack of DA signaling may differentially affect the kinematic outputs of OMR. Among the four parameters tested, directionality and latency rely on proper visual information processing in the retina and pretectum, while latency, speed, and velocity are also encoded in motor systems. In mammals, both bright and dim light have been shown to induce DA release by DA-amacrine cells (DA-AC), which improve visual and contrast sensitivity^84,85^. The zebrafish retina contains DA-AC as well as D1- and D2-like receptor-expressing cells^86,87^, and DA-AC ablation induces impairments in visual sensitivity, effects that were reversed by DA-agonist treatment^88^. Thus, we hypothesize that lack of DA-AC *th* expression will impair the proper activity of retinal cell types, leading to increased visual thresholds. Retinal Ganglion Cell (RGC) firing may only be produced after consecutive stimulation, delaying the response to stimulus onset, contributing to the increased latency in *th* mutants. Likewise, lack of DA modulation of direction-selective RGCs could also affect the extraction and transfer of motion direction to pretectal arborization fields^89^, resulting in a reduced directionality. Response latency may also involve DA modulation of supraspinal circuits controlling motor initiation. On the other hand, we suggest that the reduction in velocity and speed observed in *th* mutants may be related to dysfunction in downstream motor control circuits, which compromise the execution of the response. This is concordant with previous D1 agonist treatments performed in zebrafish larvae, which elicited faster swim responses upon OMR, based on recruitment of more primary and secondary motor neurons controlling fast versus slow swim responses^90^.

We think that our CA-deficient zebrafish model will be instrumental to address unresolved issues on DA systems development and circuit maturation, and help to distinguish between DA/NA-specific effects and other second transmitter activities of CA systems. We note that the rather subtle phenotype of the triple mutant larvae was surprising to us. We validated, based on published scRNAseq data^91^, that DAergic and adrenergic receptors are already broadly expressed in most brain regions at the 5 dpf stage (Supplementary Fig. 9). In accordance, effects of pharmacological manipulation of CA systems have been reported in 5 dpf or earlier larvae^56,61,83,92,93^. Thus, a lack of more severe phenotypes is not caused by immature CA systems.

It is interesting to compare our phenotype with invertebrate DA-deficient models. Lethal mutations in the *Drosophila* Th-encoding gene *pale* have been rescued by transgene expression of Th in hypodermal cells of the cuticle to generate viable flies devoid of DA in the brain^94,95^. These studies reveal that flies with DA-deficient brains can perform complex brain functions, but are affected in specific behaviors, and show reduced activity, locomotor deficits increasing with age, and reduced food uptake, while arousal and choice behaviors require normal DA^94^. Dopamine-deficient *Caenorhabditis elegans cat-*2 mutants are viable, but show aberrant rates of locomotion^96^. Thus, considering the effects we observe on kinematic parameters during OMR, there may be shared roles of DA in invertebrate and vertebrate control of motor activity and movement coordination.

We speculate that in our model, circuit development in the absence of CAs may lead to more pronounced compensation of loss of DA by other neuromodulators. Serotonin has been shown to pattern locomotor network activity in zebrafish^97^. In mammalian systems, DA and serotonin have also been shown to synergize or partially compensate in control of other behaviors including reward^98^. The complex interactions of DA, serotonin and other neuromodulators like endocannabinoids as well as neuropeptides^99^ may explain how potential differences in circuit development as well as intrinsic compensation mechanisms in neuromodulation may cause rather normal behavior in the absence of DA. Our model may thus also facilitate research on the interplay of neuromodulators in normal and diseased states, including Parkinson’s and depression.

## Methods

### Zebrafish maintenance, breeding, and transgenic lines

Zebrafish (*Danio rerio*) embryos and larvae were obtained by natural breeding and staged accordingly^100^. Embryos were incubated at 28.5 °C in E3 (5 mM NaCl, 0.17 mM KCl, 0.33 mM CaCl2, 0.33 mM MgSO4, pH 7.0) with 0.01% methylene blue medium at a 14:10 light/dark photoperiod. WT zebrafish are from an *AB/TL* genetic background. In addition, we used the *mitfa*^*s184*^ allele to generate a genetic background devoid of neural crest-derived melanocytes^42,101^. The generation of the zebrafish lines *th*^*m1403*^ and *th2*^*m1420*^ is described in this paper. Tyrosinase mutants (*tyr*^*tk20*^) were kindly provided by Herwig Baier^36^. Triple mutant embryos were obtained by incross of heterozygous genotypes for *th*^*m1403*^ and *th2*^*m1420*^, and homozygous for *tyr*^*tk20*^. The following transgenic lines were used *Tg(th:Gal4-VP16)*^*m1233*^, *Tg(UAS:eGFP-CAAX)*^*m1230*^^102^ and *Tg(mnx1:GFP)*^*ml2Tg*^^103^.

All zebrafish care and experiments were conducted according to the German Animal Welfare Laws under permits from the state authorities: Breeding and holding of zebrafish was approved by Regierungspraesidium Freiburg permit 35-9185.64/1.1. Animal experiments were approved by Regierungspraesidium Freiburg permits 35-9185.81/G-15/44 and 35-9185.81/G-16/96. We have complied with all relevant ethical regulations for animal use. This study was carried out in compliance with the ARRIVE guidelines.

Nomenclature: We note that by zebrafish nomenclature conventions (https://zfin.atlassian.net/wiki/spaces/general/pages/1818394635/ZFIN+Zebrafish+Nomenclature+Conventions), the triple mutant embryos, based on the chromosome locations of the loci (*th2* chromosome 4; *tyr* chromosome 15; *th* chromosome 25) should be written *th2*^*m1420/m4120*^*; tyr*^*tk20/tk20*^*; th*^*m1403/m1403*^. For easier reading, we use *th, th2, tyr* mutants or just triple mutants, and for double mutants, e.g., *th, tyr* (double) mutants. In some figures, we use *th th2 tyr* for mutant homozygous loci for the sake of space. Heterozygous combinations are designated *th* + */−, th2* + */−, tyr* + */−*.

### Generation of *th* and *th2* mutant alleles using TALEN

Loss-of-function mutations in the *th* and *th2* genes were induced using the TALEN technology^104^. TALENs were designed using the online tool *Mojo Hand* http://www.talendesign.org^105^ and assembled using the Golden Gate cloning method^106^. The clones containing the full-length TALENs were sent for sequencing, and the results were analyzed using the online program TAL Plasmids Sequence Assembly Tool (http://bao.rice.edu/Research/BioinformaticTools/assembleTALSequences.html). TALEN plasmids were linearized and then transcribed using the mMESSAGE mMACHINE® High Yield Capped RNA Transcription Kit (Thermo Fisher). TALEN mRNA pairs were injected at 100 or 200 ng/μL each into one-cell-stage WT *AB/TL* embryos and developed for one day to perform DNA extraction and PCR amplification (Figs. 1B and 2C). PCR products were then digested with TaqI or NsiI (Fig. 2B, C) for *th2*, or restricted with EcoRI for *th* (Fig. 1B). Non-cleaved bands were sent for sequencing to validate TALEN efficiency.

For the *tyrosine hydroxylase* locus (*th*), the knock-out was carried out by disrupting an EcoRI restriction site at the beginning of the open reading frame (ORF) to facilitate the analysis through restriction fragment length polymorphism (RFLP). TALENS were designed to bind to 5′-TCCGCGCGCGCGCCATCTGA-3′ on the coding and to 5′-GCAGCTCCACATCTTCCACA-3′ on the complementary strand. The spacer region was *ac****atg****cc****gaattc****aa,* carrying the restriction enzyme recognition site (*gaattc*) for EcoRI (Fig. 1A). Disruption in the restriction site results in undigested bands that demonstrate a TALEN-induced in/del mutation (Fig. 1B, EcoRI digestion, uncleaved). Sequencing of the intact bands revealed three different indel mutations. A 11 bp deletion (allele *m1404*) and a 1 bp insertion (allele *m1405*) each caused a frameshift and premature stop codon formation, while a 7 bp deletion (allele *m1403*) disrupted the native ATG initiation codon (Fig. 1C). In this study, the *th*^*+/m1403*^ line was used.

The knock-out for *tyrosine hydroxylase 2* (*th2*) was obtained by complete deletion of the coding region, from exon 1 to exon 12 (Fig. 2A). To achieve this goal, two pairs of TALEN targeting the first (*th2*-E1) and last (*th2*-E12) exon were designed. The first pair was directed toward the start of the ORF of the *th2* gene by targeting the sequences 5′-TAACTGGATATATTCA-3′ on the coding and 5′-CGGACAGTATAGCGC-3′ on the complementary strand of exon 1, while the second pair disrupted the end of exon 12 by binding to 5′-GAGGTCTAGGGTTGA-3′ and 5′-TCTCAGATGTAATAAAC-3′ on the coding and complementary strands, respectively. TALEN-binding regions include restriction sites for TaqI (exon 1) and NsiI (exon 12). TALEN activity was tested by RFLP assay. Digestion with TaqI (*th2*-E1) resulted in one thick band seen at ~250 bp, which corresponds to the overlap of the two digestion products of similar size (242 bp and 250 bp, Fig. 2B-C). Digestion with NsiI (*th2*-E12) breaks down the amplicon into two bands of 423 bp and 140 bp (Fig. 2B, C). In TALEN-injected samples, we observe both cleaved and uncleaved bands (*th2*-E1: 492 bp; *th2*-E12: 563 bp; Fig. 2C), demonstrating the efficacy of TALENs in modifying the restriction sites. Combined activity of both TALENs results in a 10 kb deletion (Fig. 2E), indicated by the presence of a 382 bp product (Fig. 2D), which is only possible after the complete deletion of the E1 to E12 sequences (Fig. 2B). In this study, the *th2*^*+/m1420*^ allele was used.

For each mutation, TALEN-injected founders (G0) were raised and identified following outcross with *AB/TL* WT fish. The resulting egg clutches were screened using PCR and restriction assay. For egg clutches with a mutant allele, siblings were raised as filial generation 1 (F1). F1 heterozygotes were crossed to *AB/TL* WT fish to establish a stable mutant line, and the sequence for each allele was determined (Fig. 1C and 2E).

### Genotyping

Genotyping of larvae was done after completion of the experiments, using DNA isolated from either the whole larvae or tail fin biopsies. Adult fish were anesthetized with MS-222 (168 mg per liter of water from the fish holding system, incubation until slowing down of operculum movement, when the tail fin was cut and fish immediately transferred to a recovery tank without MS-222) and genotyped from tail fin biopsies. After a 10 min incubation period at 98 °C, samples were digested with Lysis Buffer (stock 10x: 100 mM Tris pH 8, 500 mM KCl, 3% Tween 20, 3% NP40, 10 mM EDTA in ddH_2_O) and Protein-kinase K (10 µg/ml in PBST, A3830, AppliChem) overnight, followed by 10 min at 98 °C. Mutants for *th*^*m1403*^ were identified using primers flanking the 7 bp deletion at the EcoRI site in the first exon (forward: 5′-AAGATGTTCAATGACGGAG-3′; reverse: 5′-GTTGAAGTGGACAATGTAG-3′), followed by a restriction assay with the enzyme EcoRI*-*HF (R3101, BioLabs) (Fig. 1D). For *th2*^*m1420*^ genotyping, a common reverse primer (5′-ATGCCCCTGGAAATAGGC-3′) and two mismatch forward primers hybridizing either the WT (5′-CTTGGGGAGACTGGGACAG-3′) or mutant (5′-CTGTCGCTTGAAGCACCTG-3′) sequences were used (Fig. 2D), resulting in DNA fragments of 286 bp for WT and 396 bp for mutant alleles. Homozygous mutants for *tyr*^*tk20*^ were identified by absence of melanocyte-derived dark pigmentation.

### Whole-mount chromogenic in situ hybridization (WISH)

Digoxigenin-labeled antisense RNA probes for *hgg1* (*hatching gland 1* or *ctslb cathepsin Lb*)^54^ and *tph2 (tryptophan hydroxylase 2)*^107^ were in vitro synthesized using SP6 or T3 RNA polymerase (Thermo Scientific) following the manufacturer’s protocol. They were detected using Anti-digoxygenin-alkaline-phosphatase (0,2 µl/ml PBST, #11093279910, Roche). Whole-mount in situ hybridization (WISH) was performed as described^108^. Bleaching of melanin was performed in WT pigmented larvae shown in Fig. 5B after fixation. Larvae were immersed in a 3% H₂O₂/0.5% KOH solution until the pigmentation was fully bleached^108^. Control non-bleached WISH revealed that bleaching does not affect *tph2* WISH signal when compared to pigmented WT or *mitfa* mutants.

### Whole-mount fluorescent in situ hybridization chain reaction (HCR)

HCR^109^ was performed using the HCR^TM^ RNA-FISH Kit 3.0 (Molecular Instruments, Los Angeles, USA) according to the manufacturer’s instructions for the HCR RNA-FISH protocol for whole-mount zebrafish embryos and larvae (*Danio rerio*). Embryos were collected from (1) two separate egg clutches from *mitfa−/− tyr*+/− *th*+/− *th2*+*/−* parents, (2) *mitfa−/−*, and (3) WT *AB/TL* strain crosses and incubated in parallel under identical conditions. Larvae were fixed starting at 120 hpf in 4% PFA for 4 h at room temperature, and after fixation, the tips of the larval tails were cut for genotyping by PCR. We wanted to perform all HCR reactions in the same tube to achieve comparable stain intensities for all genotypes, and therefore needed to mark each larva based on genotyping results. WT can be distinguished from *mitfa−/−* and *tyr−/−* by pigmentation pattern. For each of the three single or double *th* and *th2* mutants (with *tyr−/−)*, based on genotype, we cut the tails at distinct lengths to later re-ID the genotypes (three levels: tail tips as cut for genotyping, cut within yolk extension, and cut within the main yolk). After dehydration, all larvae were depigmented at room temperature with 3% H_2_O_2_ in 0.5% KOH under a strong light source (Schott KL1500 electronic light source, highest intensity; 150-W halogen bulb) for 45 min^108^. For permeabilization, larvae were incubated in 10 µg/ml ProteinaseK (AppliChem, A3830) at room temperature for 2 h. The HCR probe sequences for *th* were previously published^110^. HCR probes for *th2* are listed in Supplementary Table 15. For *th*, the amplifier B4 Alexa Fluor 647 and for *th2* B5 Alexa Fluor 546 (Molecular Instruments, Los Angeles, USA) were used. For nuclear staining, 5 µg/mL DAPI (Sigma-Aldrich, A4503) was added to the amplification solution. Embryos were imaged using Zeiss LSM880 (FastAiryScan, stitching with 10% overlay, pixel dwell time 1.56 µs). AiryScan processing and stitching were performed using Zen Black 2.3 (Zeiss). The DAPI stain was used to verify cell counts, especially for low-level expressing cells. Fiji/ImageJ was used for image processing and cell counting.

### Immunofluorescence Imaging

Whole-mount immunofluorescence was performed as described^111^. Immunofluorescence staining for GFP detection was carried out using chicken anti-GFP (1:400 IgY, A10262, Life Technologies) and Alexa Fluor 488 goat anti-chicken (1:500, Fab fragment, IgG, A11039, Life Technologies). Spinal cord projecting neurons (Mauthner cells) were detected using mouse anti-neurofilament 3A10 IgG (1:20, supernatant, DSHB, 3A10)^112^ with Alexa Fluor 555 goat anti-mouse as secondary antibody (1:500, IgG, A21425, Life Technologies). Samples were counterstained with nuclear stain TOTO-3 iodide (T3604, Invitrogen) for general anatomical orientation. Anti-Th immunofluorescence was carried out with a polyclonal rabbit anti-zebrafish Th antibody^113^.

### Imaging

WISH-stained larvae were mounted in 100% glycerol and imaged with an Axioplan Microscope (10x/NA0.3 objective, Carl Zeiss, Germany) with AxioVision Software (Carl Zeiss, Germany), typically recording Z-stacks of the brain. Z-stacks were processed in ImageJ (Fiji Edition, https://imagej.net/software/fiji/) to generate minimum intensity projections. Whole-mount immunofluorescence of *Tg(mnx1a:GFP)* labeling spinal motor neurons was imaged in 80% glycerol with an Examiner microscope (20x/NA1.0 water immersion objective, Carl Zeiss, Germany) using the ZEN blue software (Carl Zeiss, Germany). Z-stacks of individual somites were analyzed with the 3D function. Motor neurons were counted at 33 h post-fertilization (hpf) in 4 somites (7, 8, 9, and 10) of 5 embryos each, while at 3 dpf 3 somites (7, 8, and 9) were counted in 5 embryos each. Immunofluorescence performed on CA tracts and axonal projections was mounted in 1% agarose containing Vectashield Mounting Medium (H-1000, Vector Laboratories), and imaged using an inverse LSM880 laser scanning confocal microscope (25x/0.8NA multi-immersion objective, Carl Zeiss, Germany).

### Dopamine ELISA

For dopamine quantification, 5 dpf larvae were euthanized with MS-222 (300 mg/liter E3 until the heartbeat stopped, followed by decapitation). Two sampling procedures were used: (1) heads of WT *AB/TL* and *tyr−/−* larvae at 20 hpf or 5 dpf were collected and immediately pooled before freezing (designated heads in bulk) in 500 µl of Homogenizing Medium (HM, 0.01 N HCl; 1 mM EDTA; 4 mM Sodium Metabisulfite; in 1× PBS), and stored at −80 °C. (2) For 5 dpf progeny obtained from single (for *th* or *th2*), double (for *th th2*) or triple (for *th, th2, tyr;* parents homozygous for *tyr*) heterozygous parents, heads were collected and stored separately (single heads). Tails were cut off for genotyping, while heads were transferred to 50 μl of HM and stored at −80 °C.

The observed fraction of specific genotypes in these crosses corresponds to the expected genetic Mendelian segregation. 50 homozygous triple mutants were obtained from 1187 pre-sorted *tyr−/−* larvae genotyped for *th* and *th2* (4.7% double homozygous, expected 1/16 = 6.25%). From *th* + */− th2 +/−* crosses, 1200 larvae were genotyped for *th* and *th2,* with 4.3% double homozygous detected (expected 6.25%). From *th*+*/−* crosses, out of 480 larvae analyzed, 24.8% *th−/−* mutants were recovered (expected 25%), and from *th2*+*/−* crosses, out of 415 larvae, 25.5% *th2−/−* mutants were recovered (expected 25%).

After obtaining the genotyping results from tail tissue, 50 heads of the specific desired genotype were pooled to a final volume of 500 μl. Samples from (1) and (2) each were manually homogenized on ice using a micropestle in a 1.5 ml reaction vial. Samples were then placed in boiling water for 3–4 min and centrifuged for 5 min at 20,000 rpm, 4 °C. Dopamine-containing supernatants were collected and stored at −80 °C. Extraction efficiency was compared using Bradford Assay (Protein Assay, #5000006, Bio-Rad®; Supplementary Table 1C). As expected, WT 20 hpf stage extracts had lower protein amounts given that the cell number in embryos at 20 hpf is much smaller than at 5 dpf. Dopamine quantification was carried out using a competitive Enzyme-Linked ImmunoSorbent Assay (Dopamine Research ELISA™ Kit, BA E-5300, LDN, Nordhorn, Germany). The ELISA is highly specific for dopamine, and for each of the kit batches used in this study, the producer LDN specified the following cross reactivities: derivatized adrenaline 0.03%; derivatized noradrenaline 0.87%; derivatized dopamine 100%; L-DOPA and Tyrosine <0.007%; Tyramine 0.13%. Extraction and acylation of dopamine were performed in a sample volume of 500 μl, followed by enzymatic conversion and dopamine ELISA. All the steps involved in dopamine quantification were done following the manufacturer’s specifications. Given that commercial kits are not designed for zebrafish extract samples, we first determined whether there is a linear relationship between the amount of extract from 5 dpf larvae and the ELISA DA readings. The ELISA linearity was validated by 1:2 dilutions of WT 5 dpf, WT 20 hpf, and *tyr* mutant samples as well as by a 1:4 dilution of WT 5 dpf. For the 20 hpf WT sample, 150 whole zebrafish larvae were used instead of 50 heads to get readings closer to the linear range of the ELISA; the calculated DA amounts were divided by three to obtain the data on DA content in 50 larvae. In addition, the ELISA was challenged by spiking 1 ng or 10 ng DA (Dopamine HCl Sigma H8502) into WT 5 dpf samples obtained from 50 WT heads in bulk (for data see Supplementary Data 1 and 2). While the spiking resulted in DA amounts higher than the linear range of 0.125 to 80 ng DA per ml, the ELISA readings were not saturated at 80 ng DA per ml and indicate that under our extract conditions, also higher DA concentrations would have been detected (Supplementary Data 1 and 2).

Comparisons were performed among samples obtained with the same extraction method. The heads in bulk samples are expected to better represent the total DA in zebrafish larval heads, because in single head preparation DA may leak out into freezing media that later was discarded to limit total sample volume to 500 µl. Indeed, comparing WT 5 dpf samples, DA amounts measured in single head extracts were smaller than those from heads in bulk. Given that protein extraction efficiency in *th th2* double and *th th2 tyr* triple mutants was similar to WT single head samples, we think that the extraction method does not significantly affect DA measurements in these samples compared to WT.

Unfortunately, we were not able to obtain the number (*n* = 3) of biological replicates required for statistical analysis for the DA ELISA assays in single heads with single mutant *th* and *th2* larvae (*n* = 2) and double or tipple mutant (*th tyr* *n* = 2; *th th2* *n* = 1; *th th2 tyr* *n* = 1) genotypes (Fig. 3B). This was caused by difficulties to obtain the number of embryos of the defined genotypes: for single mutants 200 embryos (1/4) are required for an ELISA batch of 50 heads, for double mutants 800 embryos (1/16) are required. For triple mutants, we crossed *tyr/+* or *tyr/tyr* heterozygous *th* and *th2* heterozygous fish, requiring 1600 or 800 embryos, respectively. We in addition observed that double heterozygous fish, and more pronounced triple heterozygous fish, grow with a distorted male-to-female fish ratio, and tend to produce fertilized embryos less frequently and in smaller numbers; however, we have not been able to collect sufficient data to validate these observations. Pooling of single head material from multiple experimental days tends to contribute to deterioration of sample quality (see above on comparison of measurements from bulk head versus single head isolation samples), and therefore was not an option to increase the number of biological replicates.

### Heart rate

Heart rate (HR) and heart rate variability (HRV) were obtained as described^114,115^. Briefly, heartbeat was recorded at 3 dpf between 76 and 81 h post-fertilization in non-anaesthetized and free-moving larvae. Video acquisitions were done at 35 frames per second (fps) using a USB 3.1 monochrome camera (DMK 37BUX250, Imaging Source, Germany) equipped with the IC Capture 2.4 software. Recordings were performed in larvae at control temperature (28 °C) and at four thermal challenges (20 °C, 24 °C, 32 °C, and 36 °C). Larvae were all raised at 28 °C, transferred to the required experimental temperature, given a 1 min period of acclimatization, and subsequently heartbeat was recorded on video for 1 min. Video files were saved in AVI (Audio Video Interleave) format and decompressed using FFmpeg^116^. All spontaneous motion, such as pectoral fin and gill movements, was not considered for the final analysis. Video frames were imported into ImageJ. Heart rate was extracted with ImageJ by selecting a region in the heart^114^ and using the plot Z-axis profile function in ImageJ to reveal the pixel intensity changes over time produced by every heartbeat, while HRV was calculated as the root mean square of the successive differences between heartbeat intervals (RMSSD)^115^.

### Hatching phenotype

Hatching gland cell (HGC) retention was assessed in larvae from crosses of *th*+*/−, th2*+*/−, tyr−/−* parents. At 3 dpf, the larvae were divided into two groups based on the presence (HGC+) or absence (HGC−) of HGCs in the pericardial membrane (Fig. 7A). About 25% of the larvae still had HGCs at 3 dpf (HGC+ phenotype) and were selected, while a similar number of HGC− larvae were randomly chosen from the remaining three-quarters of larvae. After genotyping, the relation between HGC phenotype and genotype was examined. At 5 dpf, the abundance of granule-containing cells in the pericardial membrane was classified into high (over 80% of the pericardial membrane still containing HGC), low (less than 20% of the pericardial membrane containing HGC), and no-retention (no HGC cells observed) (Fig. 7D). The HGC presence was assessed using a binocular stereomicroscope (Leica MZ12, Germany).

### Transcriptomic analyses

scRNAseq data from 5 dpf zebrafish brain and eye cells^91^ were downloaded from GEO: GSE158142 (https://www.ncbi.nlm.nih.gov/geo/query/acc.cgi?acc=GSE158142, GSE158142_zf5dpf_cc_filt.cluster.rds, downloaded on 05/11/2020) and analyzed by the Seurat package (v4.3.0)^117^. Seurat object was updated from v2.X to v3.X using the SeuratObject::UpdateSeuratObject (v4.1.3) function. Clustering and annotation were retained from the original data from Raj et al., 2020. The average expression levels for the respective genes were calculated using Seurat::AddModuleScore. FItSNE plots were generated using Seurat::DimPlot and Seurat::FeaturePlot functions. The expression of CA Receptors in HGCs was investigated using the online resource Daniocell (https://daniocell.nichd.nih.gov/index.html, v1.1, 02/01/2024)^39^. HGC clusters (axia_15, axia_18, axia_20) were confirmed by expression of *cathepsin Lb (ctslb)* and *hatching enzyme 1* (*he1.1)*.

### Spontaneous locomotor activity

Video recordings of spontaneous swimming were performed under white light at a frame rate of 30 fps for 1 h in the Noldus DanioVision observation chamber (model number DVOC004x/T; Noldus, Wageningen, Netherlands) at 28.5 °C in a 24-well format. Visual recognition of unpigmented larvae in the videos was improved by measuring locomotor activity on a 24-well plate lid (lid with circular lines for each well; Cellstar, Grainer Bio-One, Germany), instead of the deep well bottom parts of the plastic dishes. The lines on the lid separating the wells were made into circular barriers by application of 3% agarose outside the circles to avoid mixing of larvae. The lid wells had a 12 mm diameter and 7 mm depth and were each filled with 800 μl E3 medium. Videos were recorded using Noldus DanioVision standard settings and tracked using the software Ethovision XT14.

### Optomotor response (OMR)

OMR assays were performed in a custom-made multi-chamber plate containing 6 rectangular rails of 1 cm width and 7 cm length (Fig. 9B). The transparent plate was positioned directly on a 4 K 15,6 Zoll Portable Monitor (3840 * 2160 pixels; Thinlerain Shenzhen Xinliangyuan Technology Co., Ltd., Shenzhen, CN). The black and white OMR gratings were generated with the Visual Stimulator made by Prof. Michael Bach (https://michaelbach.de/sci/stim/movingGrating/index.html^118^.) using maximum contrast. Bright light for pre-stimulus conditioning was followed by presentation of moving gratings from below to free-swimming larvae. For assessing OMR, *tyr* phenotype zebrafish larvae were acclimatized to bright light for 30 min in order to avoid visual impairments related to Sdy/Tyrosinase depletion^36^. Movement tracks were manually generated from the video data using the Manual Tracking plugin in ImageJ. The animals were classified as responders (engage OMR), non-responders (move randomly), or non-moving (do not move at all). For each responder, the OMR was individually measured, and trajectory, displacement, and time were extracted from the tracks. The measurement started with the onset of the stimulus and ended when the larva reached the end of the trail. In order to make the results comparable, raw variables were normalized considering the ratios among them, resulting in directionality (displacement/trajectory), speed (trajectory/time), and velocity (displacement/time). Latency was measured as the time delay between stimulus onset and optic-flow oriented movement.

### Statistics and reproducibility

Statistical analysis and plots were done using the R package ggstatsplot^119^. Data distribution was tested using the Shapiro–Wilk test for small sample sizes (*n* < 20) and the Kolmogorov–Smirnov normality test for larger sample sizes (ks.test). Parametric and non-parametric statistical tests were selected according to data distribution, followed by post-hoc pairwise comparisons. The function ggbetweenstats was used for statistical testing of unpaired samples as well as for effect size estimations. For categorical data, the function ggbarstats was used. Differences among qualitative variables were assessed using Pearson’s chi-squared (χ^2^) test. Two-way ANOVA was performed to evaluate the effect of temperature and genotype on heart function. Since ggstatsplot does not support two-way ANOVA, the test was performed using the functions aov and Anova with Type-III sums of squares for unbalanced designs (different sample sizes within each level). TukeyHSD was performed post-hoc for multiple-pairwise comparisons. Statistical significance was set at *p* < 0.05. Data presented as violin plots depict data distribution from maximum to minimum values, as well as boxplots showing median, upper and lower quartiles, while bar plots show mean and standard deviation (SD). For outlier analysis, data values differing significantly from the observations were identified according to Tukey’s rule using an outlier coefficient of 1.5. To reduce the probability of type II error, in Supplementary Fig. 8 outliers were eliminated from the analysis. All experiments and analysis were performed blinded to the genotype. Further statistical details, including tests and sample sizes, are presented in the figure legends.

### Biological materials

The mutant alleles *th*^*m1403*^ and *th2*^*m1420*^ generated in this study can be obtained from the corresponding author, W.D. Upon publication, both alleles will also be submitted to ZIRC Zebrafish International Resource Center, Eugene, USA.

### Reporting summary

Further information on research design is available in the Nature Portfolio Reporting Summary linked to this article.

## Supplementary information

Supplementary Information

Description of Additional Supplementary Materials

Supplementary Data 1-8

Reporting Summary

## Data Availability

All numerical data are provided in supplementary data and supplementary tables, together with analyses. Source data underlying Fig. 3 are provided in Supplementary Data 1–3 and Supplementary Tables 1 and 2. Source data underlying cell counts for the experiment shown in Fig. 4 are provided in Supplementary Data 4 and 5 and Supplementary Tables 3 and 4. Source data underlying cell counts for the experiment shown in Fig. 6 are provided in Supplementary Tables 5 and 6. Source data underlying Fig. 7B are provided in Supplementary Table 7. Source data underlying Fig. 7E are provided in Supplementary Table 8. Source data underlying Fig. 8 are provided in Supplementary Data 6-7 and Supplementary Tables 9 and 10. Source data underlying Fig. 9A are provided in Supplementary Table 12. Source data underlying Fig. 9C are provided in Supplementary Table 13. Source data underlying Fig. 10 are provided in Supplementary Data 8 and Supplementary Table 14. Source data underlying Supplementary Fig. 3 are provided in Supplementary Data 1 and 2. Source data underlying Supplementary Fig. 4 are provided in Supplementary Data 4 and 5 and Supplementary Tables 3 and 4. Source data underlying Supplementary Fig. 6 are provided in Supplementary Data 6 and 7 and Supplementary Tables 9 and 10. Source data underlying Supplementary Fig. 7 are provided in Supplementary Table 11. Source data underlying Supplementary Fig. 8 are provided in Supplementary Data 8 and Supplementary Table 14. Original uncropped images of all gel images are provided in Supplementary Fig. 10. All primary image data, videos, and behavioral tracking are available on request from the corresponding author, W.D.
