# Supplementary material for: A genetic model for development, physiology and behavior of catecholamine-deficient zebrafish larvae
Source: Commun Biol. 2026 Aug 5;9:1045. doi: 10.1038/s42003-026-10732-9 (PMC13444079; doi:10.1038/s42003-026-10732-9)
Supplement: Supplementary file 1 [file 42003_2026_10732_MOESM2_ESM.docx]

**Description of Additional Supplementary Files**

The Additional Supplementary Data are assembled into one EXCEL File (file name 42003_2026_10732_MOESM3_ESM.xlsx) including 8 separate spreadsheet sheets that each contain one Supplementary Data item:

**Supplementary Data** **name:** Supplementary Data 1

**Description:** Dopamine ELISA measurements Series A (related to Figure 3 and Supplementary Figure 2 Dopamine ELISA values)

**Supplementary Data** **name:** Supplementary Data 2

**Description:** Dopamine ELISA measurements Series A (related to Figure 3 and Supplementary Figure 2 Dopamine ELISA values)

**Supplementary Data** **name:** Supplementary Data 3

**Description:** Bradford Protein Assays for Dopamine ELISA Series A and calculation of Dopamine content in pMol DA per mg Protein (related to Figure 3 and Supplementary Figure 2)

**Supplementary Data** **name:** Supplementary Data 4

**Description:** *th* expressing cell counts statistical analysis (related to Figures 4 and S3)

**Supplementary Data** **name:** Supplementary Data 5

**Description:** *th2* expressing cell counts statistical analysis (related to Figures 4 and S3)

**Supplementary Data** **name:** Supplementary Data 6

**Description:** HR temperature dependence statistical analysis (related to Figure 8)

**Supplementary Data** **name:** Supplementary Data 7

**Description:** HRV temperature dependence statistical analysis (related to Figure 8)

**Supplementary Data** **name:** Supplementary Data 8

**Description:** Optomotor response kinematic parameters (related to Figure 10)
