# Supplementary material for: A genetic model for development, physiology and behavior of catecholamine-deficient zebrafish larvae
Source: Commun Biol. 2026 Aug 5;9:1045. doi: 10.1038/s42003-026-10732-9 (PMC13444079; doi:10.1038/s42003-026-10732-9)
Supplement: Supplementary file 1 [file 42003_2026_10732_MOESM1_ESM.pdf]

Supplementary Information for:

A genetic model for development, physiology and behavior of catecholamine-deficient zebrafish larvae

Susana Paredes-Zúñiga, Rebecca Peters, Kristine Østevold, Gerard Arrey, Johanna Wehrle, Dennis Frank, Johannes Oswald, Florian Veit, Theresa Schredelseker, Sandra Glauben, Jochen Holzschuh and Wolfgang Driever*

*Corresponding Author: University of Freiburg, Biology 1, Hauptstrasse 1, D-79104 Freiburg, GERMANY, email: driever@biologie.uni-freiburg.de

Inventory of Supplementary Materials

Supplementary Figures (Pages 3 – 16)

Supplementary Figure 1. Catecholamine biogenesis and catecholamine systems in zebrafish

Supplementary Figure 2. Dopamine ELISA. (A) comparison of data obtained from heads collected in bulk or single, (B) Estimation of relative contributions of individual genes to total dopamine.

Supplementary Figure 3. Cell counts of (A) *th* and (B) *th2* expressing CA neurons in two strains present in genetic background of CA system mutations (wildtype ABTL and *mitfa* mutant) as well as *tyr* combined with *th* and/or *th2* mutants in *mitfa* background.

Supplementary Figure 4. Expression of HGC markers and selected CA Receptors in HGC clusters

Supplementary Figure 5. HR and HRV of *AB/TL* WT controls and *tyr* mutant larvae

Supplementary Figure 6. Comparison of HR and HRV changes at different temperatures for specific *tyr, th* and *th2* mutant combinations

Supplementary Figure 7. Spontaneous locomotion of *AB/TL* WT controls and *tyr* mutant larvae

Supplementary Figure 8. OMR kinematic parameters in CA depleted larvae (with outlier analysis)

Supplementary Figure 9. Expression of CA receptors in 5dpf brain cells

Supplementary Figure 10. Uncropped images of gel electrophoresis images from Figures 1 and 2

Supplementary Tables (Pages 17-31)

Supplementary Table 1: Dopamine ELISA results for WT and *tyr* mutants (related to Figure 3A)

Supplementary Table 2: Comparisons of wild type samples and *tyr* mutants DA ELISA; statistics (related to Figure 3A)

Supplementary Table 3: *th* expressing cell counts (related to Figures 4 and S3)
Supplementary Table 4: *th2* expressing cell counts (related to Figures 4 and S3)
Supplementary Table 5: Figure 6B cell counts 33 hpf
Supplementary Table 6: Figure 6B cell counts 3 dpf
Supplementary Table 7: Figure 7B HGC phenotypes 3 dpf
Supplementary Table 8: Figure 7E levels of HGC retention among genotypes from 3 dpf to 5 dpf
Supplementary Table 9: Figure 8HR and HRV temperature dependence data
Supplementary Table 10: Figure 8 HR and HRV temperature dependence statistical analysis summary
Supplementary Table 11: Supplementary Figure 7 locomotor activity
Supplementary Table 12: Figure 9A locomotor activity
Supplementary Table 13: Figure 9C optomotor response
Supplementary Table 14: Figure 10 optomotor response two-sample Kolmogorov-Smirnov test
Supplementary Table 15: Methods: Sequence of oligos used for *th2* HCR in situ detection

Supplementary DATA

Provided in separate MS Word EXCEL file (Paredes-Zuniga_Supplementary_DATA_01_to_08). Each Supplementary Data are provided in a separate tab of the EXCEL file.

Supplementary DATA 1: Dopamine ELISA measurements Series A (related to Figure 3 and Supplementary Figure 2 Dopamine ELISA values)
Supplementary DATA 2: Dopamine ELISA measurements Series A (related to Figure 3 and Supplementary Figure 2 Dopamine ELISA values)
Supplementary DATA 3: Bradford Protein Assays for Dopamine ELISA Series A and calculation of Dopamine content in pMol DA per mg Protein (related to Figure 3 and Supplementary Figure 2)
Supplementary DATA 4: *th* expressing cell counts statistical analysis (related to Figures 4 and S3)
Supplementary DATA 5: *th2* expressing cell counts statistical analysis (related to Figures 4 and S3)
Supplementary DATA 6: HR temperature dependence statistical analysis (related to Figure 8)
Supplementary DATA 7: HRV temperature dependence statistical analysis (related to Figure 8)
Supplementary DATA 8: Optomotor response kinematic parameters (related to Figure 10)

Supplementary References (Page 32-33)

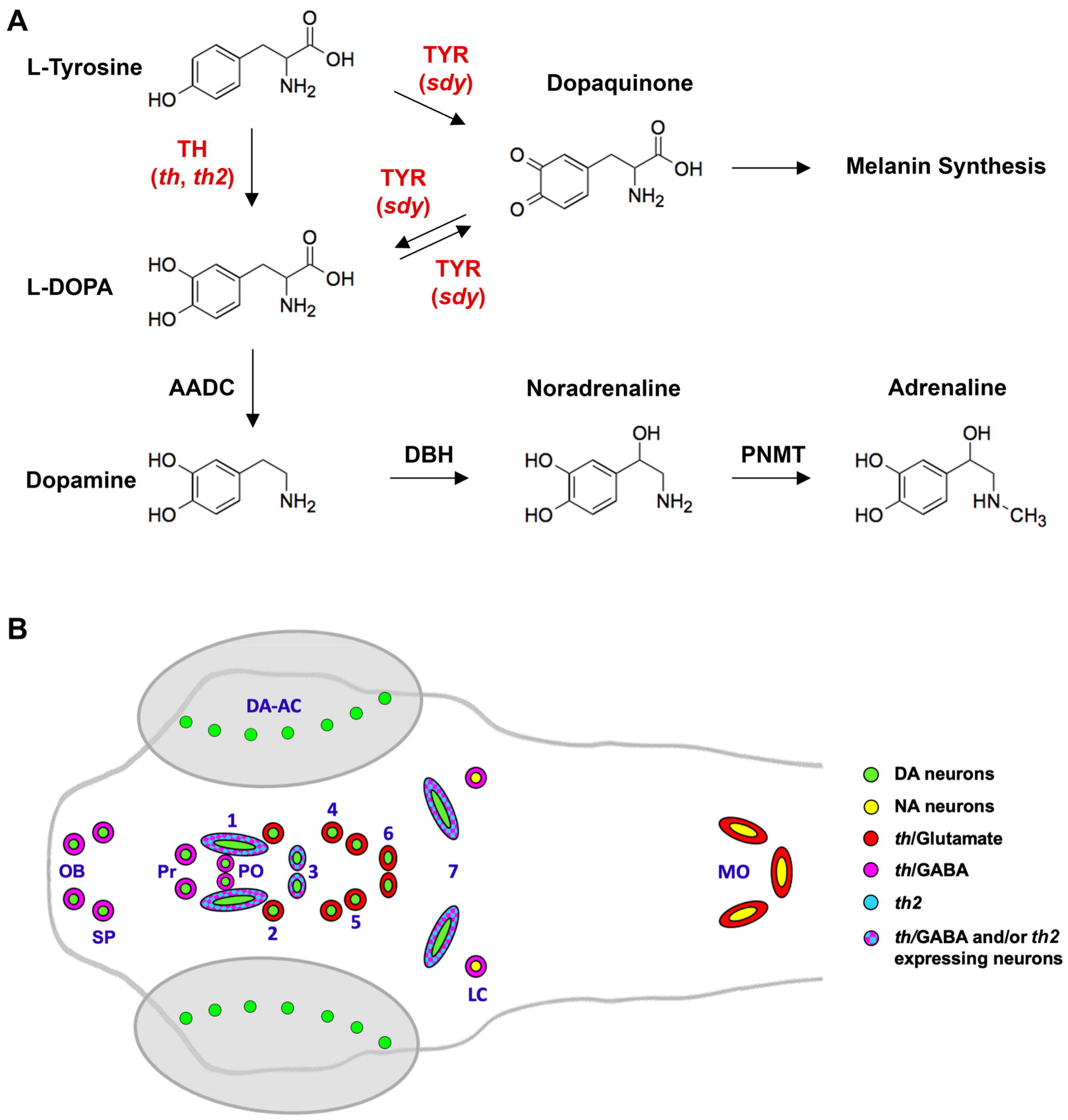

Supplementary Figure 1. Catecholamine biogenesis and catecholamine systems in zebrafish
(A) Enzymatic pathways of catecholamine synthesis. AADC: Aromatic L-Amino Acid Decarboxylase; DBH: Dopamine β-hydrolase; L-DOPA: L-3,4-dihydroxy-phenyalanine; PNMT: Phenylethanolamine-N-methyltransferase; TH: Tyrosine hydroxylase (*th* and *th2* loci in zebrafish); TYR: Tyrosinase (*sdy* locus in zebrafish). Modified from[1].
(B) Catecholamine systems in zebrafish larvae. Anatomical scheme of CA systems in the 3-5 days post fertilization (dpf) larval zebrafish brain. Dorsal view, anterior left. CA systems expressing *th* are shown using the nomenclature developed by Rink et al.[2,3]. The DA systems have evolved significantly and show both distinct and shared features with the mammalian systems[4,5]. Compared to mammalian DA systems, the diencephalospinal DA systems (A11 in mammals, DC 2,4,5 and 6 in the zebrafish posterior tuberculum; DC = DA Cluster, figure shows numbers only), the prethalamic system (A13 versus DC1), olfactory system

(A16 versus OB), and amacrine retinal system (A17) are considered largely conserved. In zebrafish, the preoptic system (A15 versus PO) is complemented by the optic recess region DA system (ORR). The hypothalamic A12 and A14 systems develop divergently in the zebrafish, which have mammillary (DC3) and tuberal (DC7) hypothalamus DA systems[6]. Zebrafish, contrary to mammals, have DA systems in the pretectum (Pr)[7] and extended medial amygdala of the subpallium (SP)[8]. Furthermore, mesdiencephalic DA clusters (A8, A9 and A10 in mammals) are absent in zebrafish, likely due to secondary loss in teleost evolution, given that they are found in lungfish[9], and very few non-functional remnants have also been detected in zebrafish[10]. The hindbrain (LC locus coeruleus and MO medulla oblongata) and peripheral (carotid body and sympathetic ganglia; not shown) NA systems are largely conserved[7,11,12].
The CA clusters DA (green) and NA (yellow) have initially been described based on *th* expression. By 5 dpf, *th2* (cyan) expression domains occur in the hypothalamic posterior and lateral recess, the paraventricular organ and preoptic region[13,14]. GABAergic (magenta) or glutamatergic (red) second transmitters are indicated. Abbreviations: AC: amacrine cells of retina; OB: olfactory bulb; SP: subpallium; Pr: pretectum; PO: Preoptic system; 1-7: DC clusters 1 to 7; LC: locus coeruleus; MO: medulla oblongata.

A

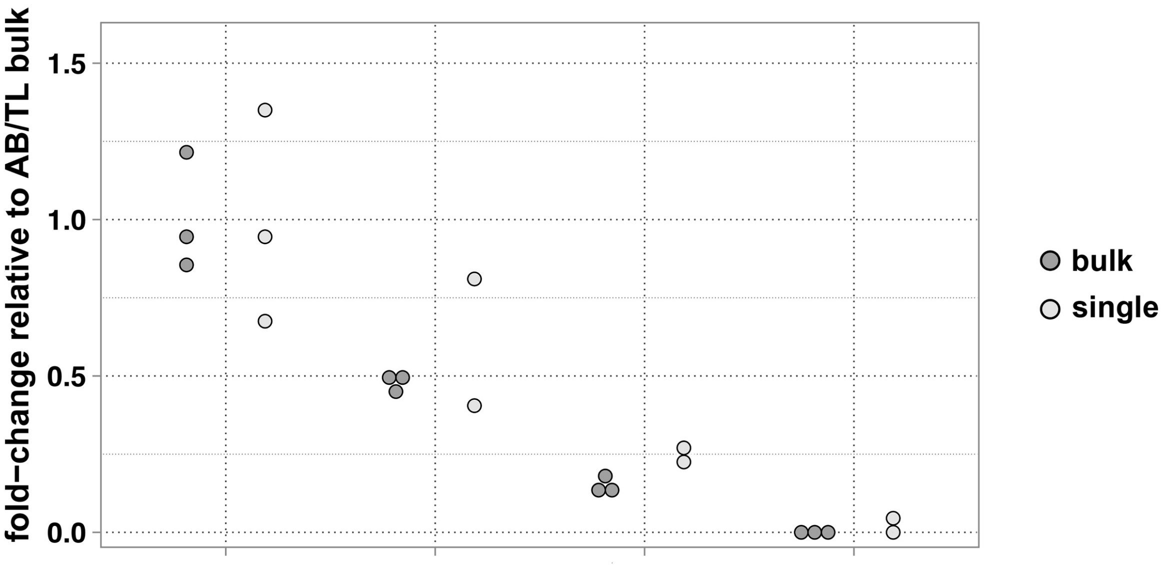

**Strain**	***AB/TL***							
**Age**	**5 dpf**						**20 hpf**	
**Collection**	**● bulk heads versus ○ single heads**							
**Dilution**	**–**		**1/2**		**1/4**		**–**	
**n**	**3**	**3**	**3**	**2**	**3**	**2**	**3**	**2**
**% of WT**	**100**		**48**	**62**	**15**	**26**	**1.6**	**4**

Supplementary Figure 2. Dopamine ELISA.

(A) Comparison of DA ELISA data obtained from heads collected in bulk and single heads. The sample numbers (n) in the table give the number of biological replicates for each condition. For data see Supplementary Data 1 - 3.

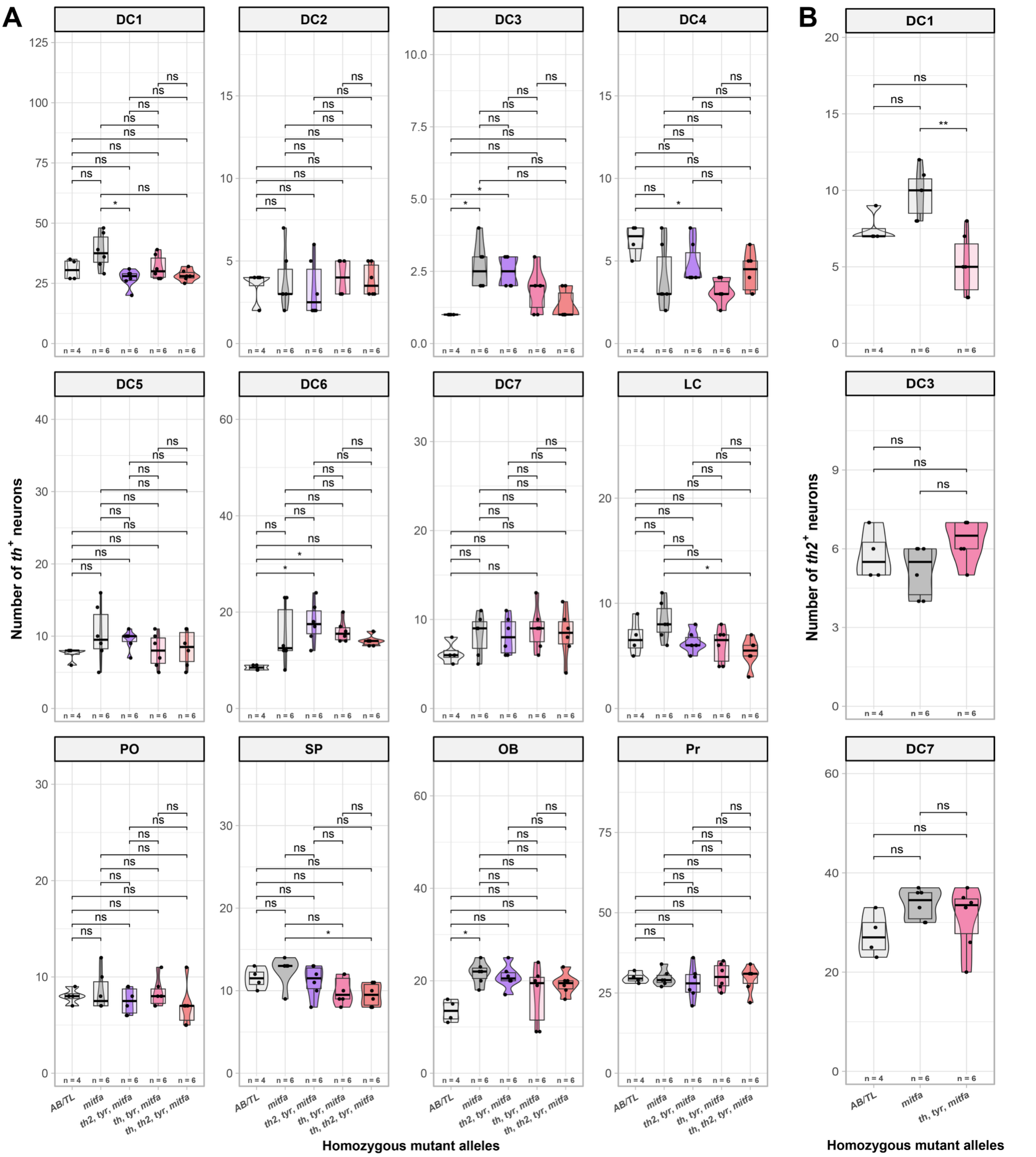

Supplementary Figure 3. Cell counts of *th* and *th2* in the catecholaminergic cell clusters.

Cell counts from experiment shown in Figure 4 for (A) *th* and (B) *th2* expressing CA neurons in two reference strains (wildtype AB/TL and *mitfa* mutant) as well as *tyr* combined with *th* and/or *th2* mutants in *mitfa* mutant background (A: Supplementary Table 3; B: Supplementary Table 4). Brains were fixed at 120 hpf, and cells counted separately for left and right halves of the larval, and numbers represent neurons per hemisphere. For statistical analysis, Kruskal-Wallis test and Dunn's post-hoc comparisons (Bonferroni-adjusted) was used (for A see Suplementary Data 4, and for B see Supplementary Data 5; ns: p-value >

0.05; *: p-value < 0.05; **: p-value < 0.001). For nomenclature and anatomical locations of CA clusters see Supplementary Figure 1B. Boxes represent the interquartile range (25th–75th percentiles, 50% of the data), the central line corresponds to the median and the vertical lines show the spread of the remaining data.

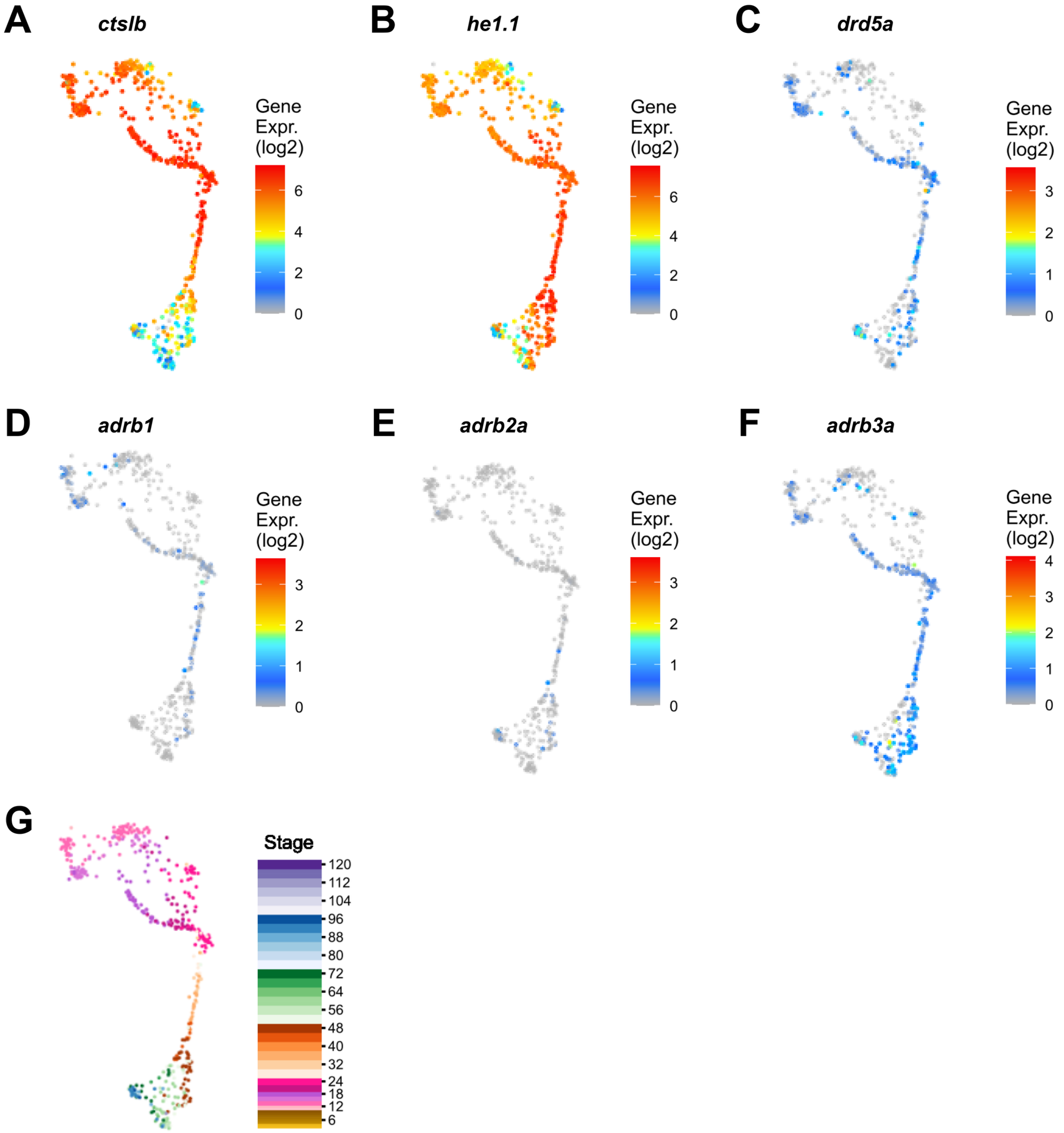

Supplementary Figure 4. Expression of HGC markers and selected CA Receptors in HGC clusters
Expression of HGC markers (A,B) and selected CA Receptors (C-F) in HGC clusters of scRNAseq data from Daniocell online resource (https://daniocell.nichd.nih.gov/index.html, v1.1)[15,16]. The analysis is based on 38,371 cells for stages 3,3 to 12 hpf, and 451,315 cells for stages 14 to 120 hpf. Data are from 3.3–120 hpf (G) whole-animal AB/TL zebrafish. Only cell mapped in the Daniocell resource to the axial mesoderm clusters axia_15, axia_18, and axia_20 are shown. In A-F, the color scale represents the log2 fold change of mean expression in relation to all cells analysed at this stage (for details see Daniocell website and publications)[15,16].

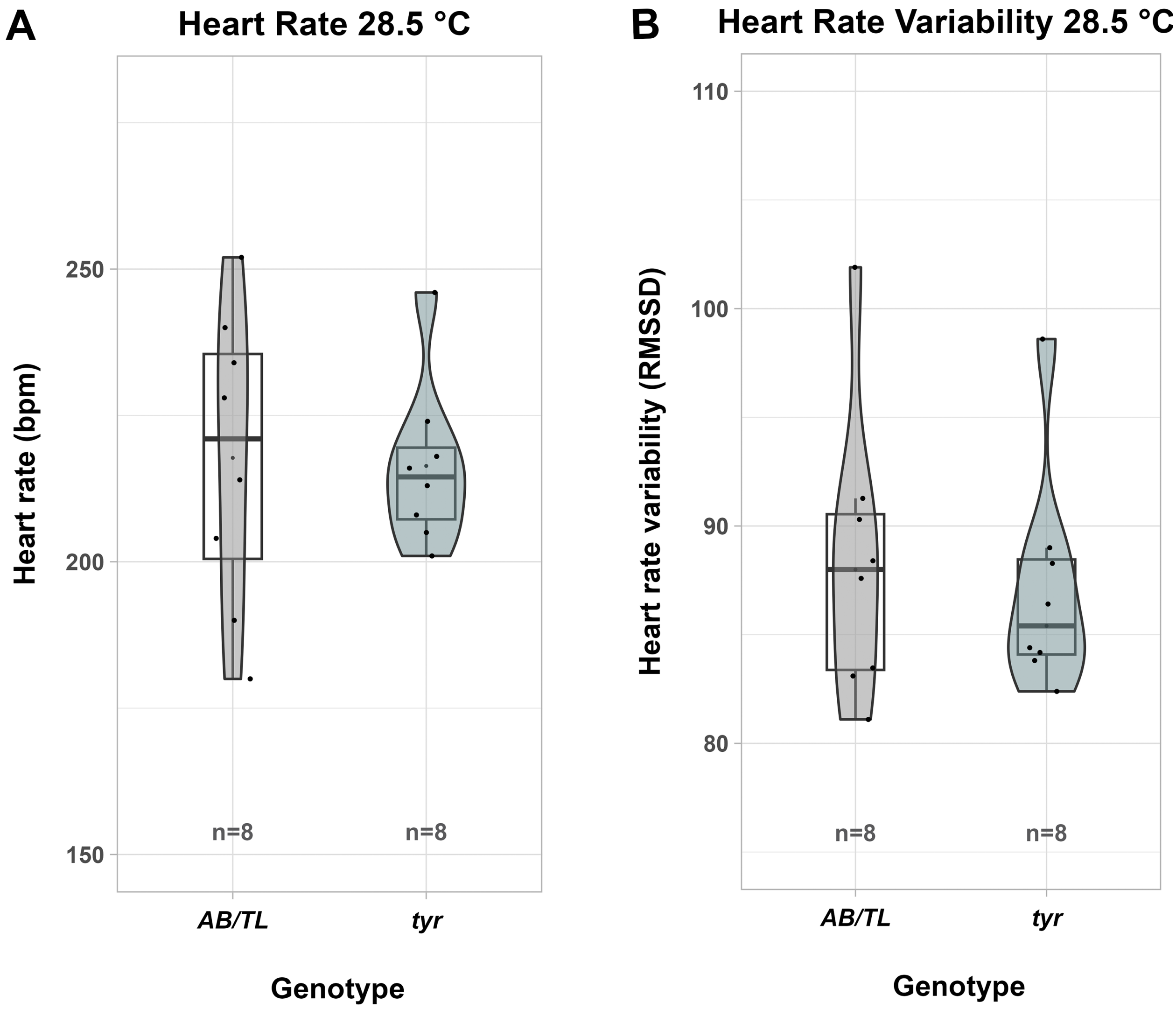

Supplementary Figure 5. HR and HRV of *AB/TL* WT controls and *tyr* mutant larvae
Relates to Main Figure 6. HR and HRV are not significantly different in *tyr* mutants compared to AB/TL WT larvae at 28.5°C. (A) For HR statistical analysis was performed using two-tailed Welch's t-test (t-Welch (11) = 0.13, $p = 0.90$, effect size (Hedges's g) = 0.06, CI 95% [-0.85, 0.97]). (B) For HRV two-tailed Mann-Whitney test was used (W = 35, $p = 0.79$, effect size (r (rank biserial)) = 0.09, CI 95% [-0.45, 0.59]). Boxes represent the interquartile range (25th–75th percentiles, 50% of the data), the central line corresponds to the median and the vertical lines show the spread of the remaining data.

**HR (bpm)**	
AB/TL	***tyr***
204	224
190	216
180	213
214	205
240	201
228	208
252	246
234	218

**HRV (RMSSD)**	
AB/TL	***tyr***
91,27	82,39
90,3	98,6
101,9	88,28
88,4	83,81
83,47	86,41
87,59	89
83,1	84,4
81,1	84,18

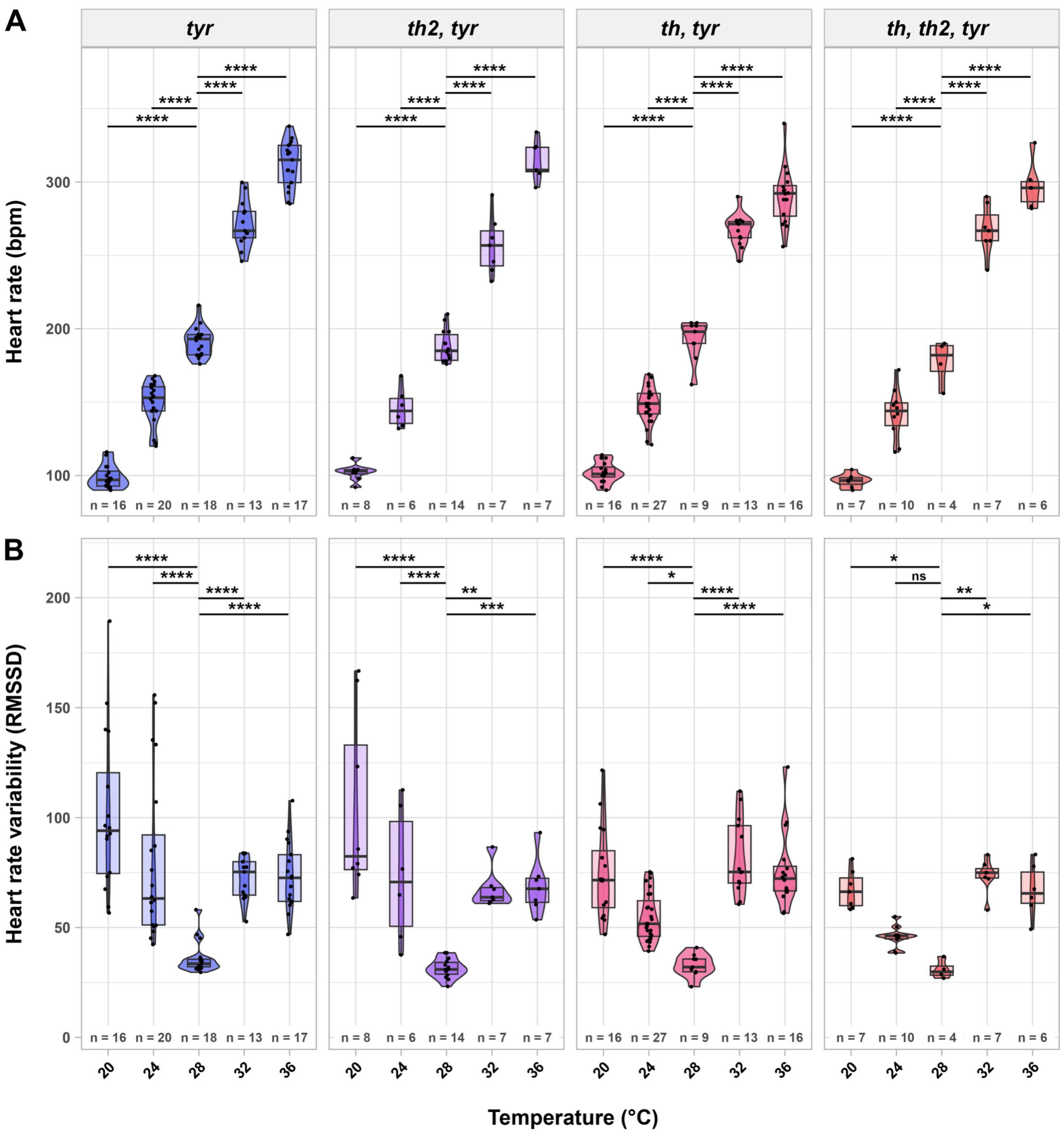

Supplementary Figure 6. Comparison of HR and HRV changes at different temperatures for specific *tyr*, *th* and *th2* mutant combinations

Relates to Main Figure 7. (A) HR and temperature appear positively related for all genotypes. (B) HRV increases with temperature challenges. Significances were calculated comparing each temperature with 28°C. Statistical analysis was performed using two-way ANOVA with Tukey's multiple pairwise-comparisons test (See Supplementary Data 6 and 7). *: p-value < 0.05; **: p-value < 0.001; ***: p-value < 0.0001; ****: p-value < 0,00001. Boxes represent the interquartile range (25th–75th percentiles, 50% of the data), the central line corresponds to the median and the vertical lines show the spread of the remaining data.

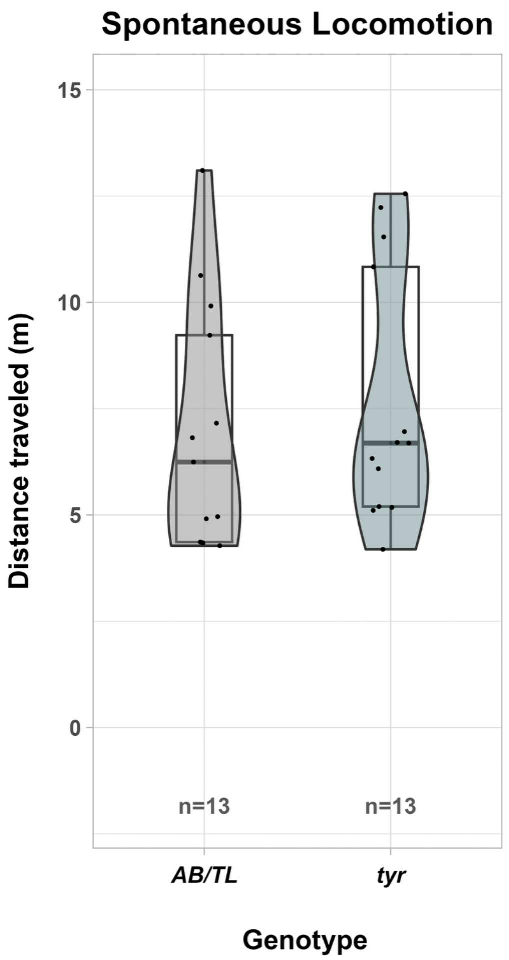

Supplementary Figure 7. Spontaneous locomotion of *AB/TL* WT controls and *tyr* mutant larvae
Relates to Main Figure 9. Spontaneous locomotor activity measured as distance traveled in 1 hour (hr) is not significantly different in *tyr* mutants compared to AB/TL control larvae (Supplementary Table 11). Statistical analysis was performed using two-tailed Mann-Whitney test (W = 68, p = 0.41, effect size (r (rank biserial)) = -0.20, CI 95% [-0.57, 0.25]). Boxes represent the interquartile range (25th–75th percentiles, 50% of the data), the central line corresponds to the median and the vertical lines show the spread of the remaining data.

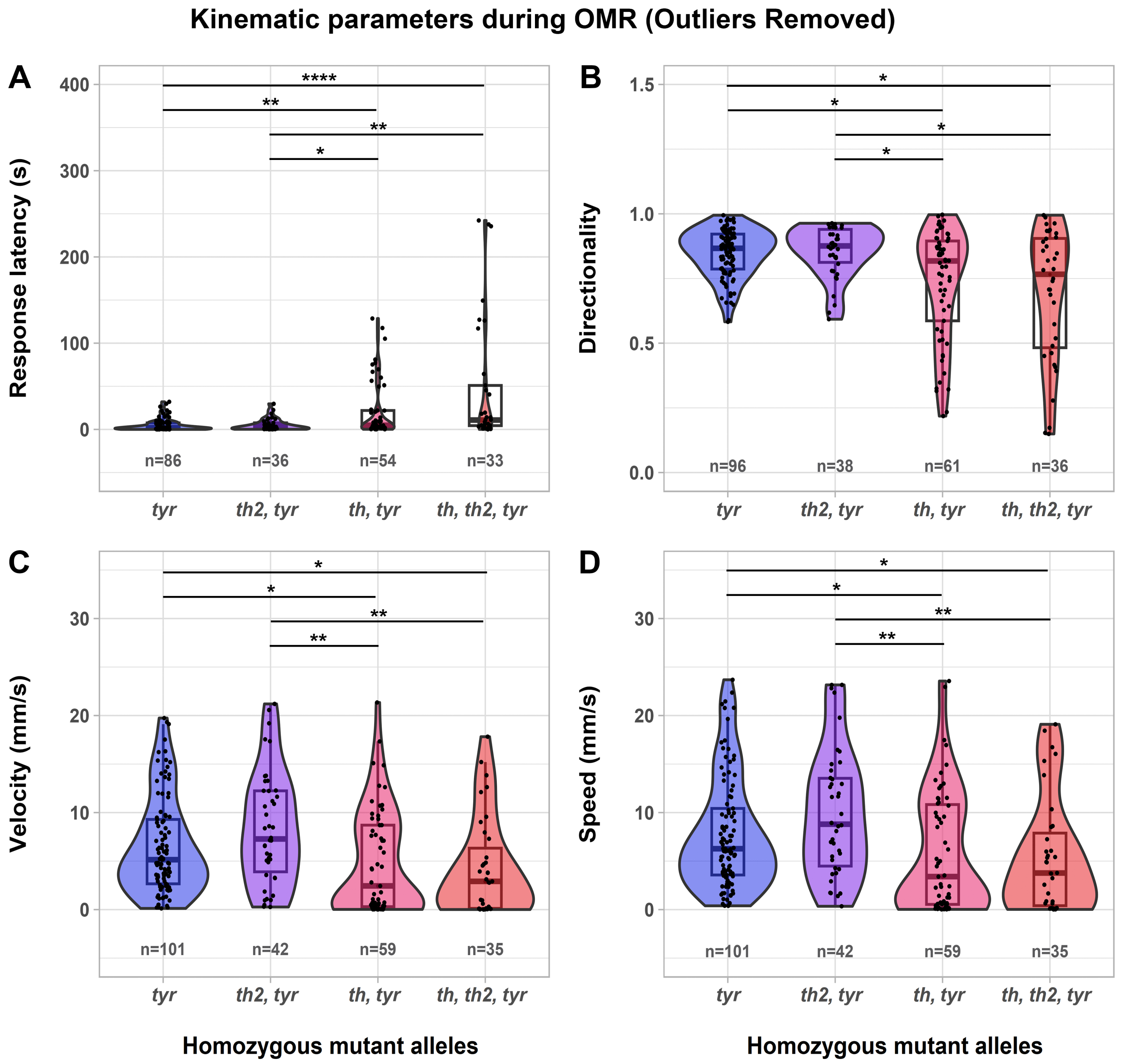

Supplementary Figure 8. OMR kinematic parameters in CA depleted larvae (with outlier analysis) (A) Latency in OMR initiation ($\chi2$ Kruskal-Wallis (3) = 25.33, p = 0.00001, effect size (ε^2) = 0.12, CI 95% [0.05, 1.00]), (B) Directionality ($\chi2$ Kruskal-Wallis (3) = 15.78, p = 0.00126, effect size (ε^2) = 0.07, CI 95% [0.04, 1.00]), (C) Velocity (vector) ($\chi2$ Kruskal-Wallis (3) = 21.13, p = 0.00009, effect size (ε^2) = 0.09, CI 95% [0.04, 1.00]), (D) Speed (scalar) ($\chi2$ Kruskal-Wallis (3) = 20.19, p = 0.000155, effect size (ε^2) = 0.09, CI 95% [0.03, 1.00]). Statistical analysis was performed using Kruskal-Wallis non-parametric test with Dunn´s pairwise comparisons and Holm p adjustment method (Supplementary Table 14). ns: p-value > 0.05; *: p-value < 0.05; **: p-value < 0.001; ***: p-value < 0.0001; ****: p-value < 0.00001. For a better visualization, outliers are not shown in the figure, but highlighted in the Supplementary Excel file (Supplementary Data 8). Outlier analysis supports that *th* is required for normal kinematic performance. Boxes represent the interquartile range (25th–75th percentiles, 50% of the data), the central line corresponds to the median and the vertical lines show the spread of the remaining data.

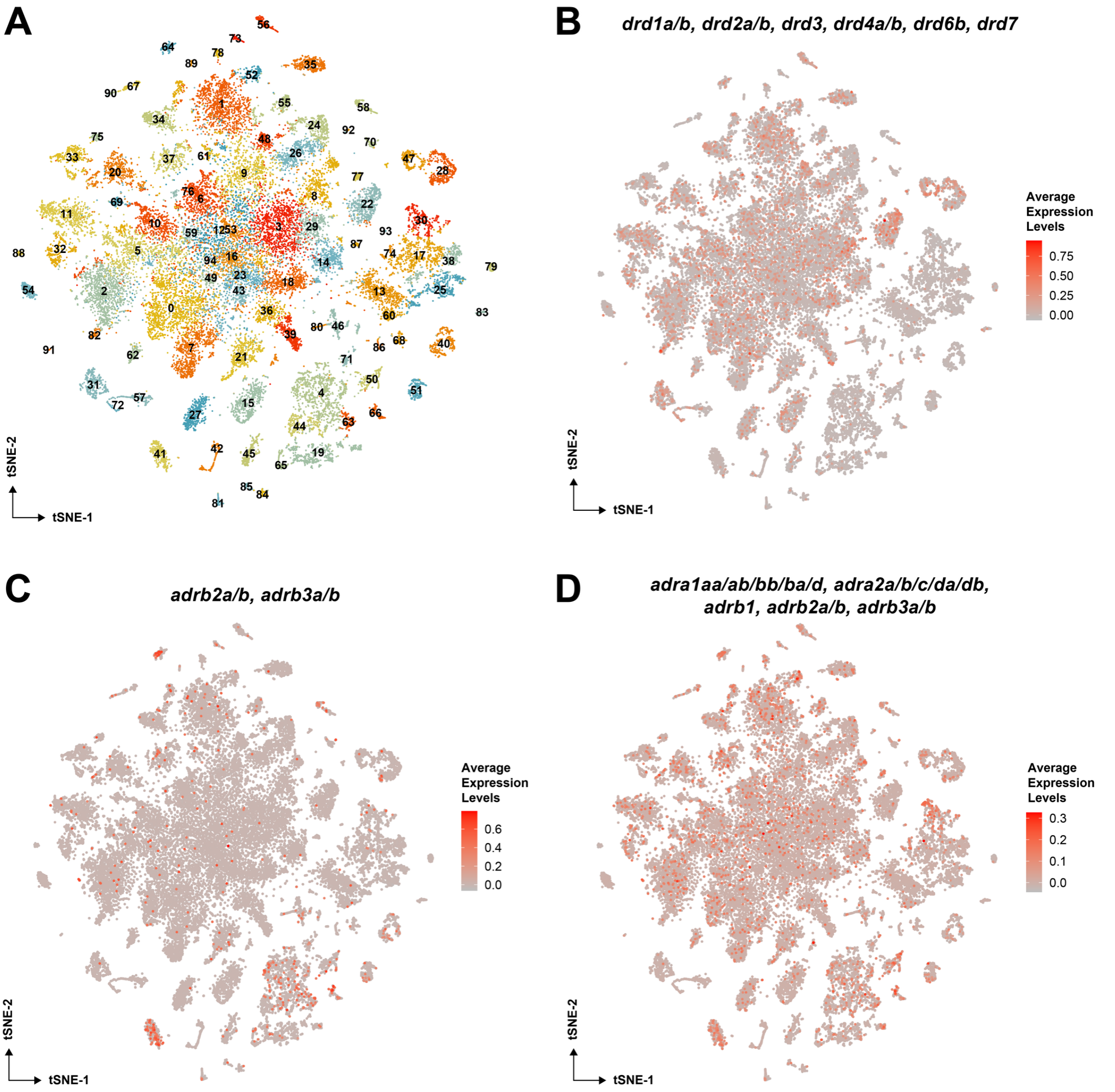

E

- 0 optic tectum (gabaergic)
- 1 ventral forebrain (dienc, hyp, poa; gabaergic)
- 2 diencephalon (tuberculum; gabaergic)
- 3 dorsal diencephalon (thalamus)
- 4 cartilage
- 5 mid-hind boundary (gabaergic)
- 6 neurons (glutamatergic)
- 7 optic tectum (gabaergic)
- 8 neurons
- 9 neurons
- 10 neurons (glutamatergic)
- 11 hindbrain/cranial nerve
- 12 neurons (glutamatergic)
- 13 progenitors
- 14 dorsal diencephalon (thalamus)
- 15 retina (cone bipolar cells)
- 16 neurons
- 17 progenitors
- 18 midbrain
- 19 cartilage
- 20 hindbrain
- 21 retina (amacrine, gabaergic)
- 22 diencephalon
- 23 midbrain
- 24 telencephalon (pallium), glutamatergic
- 25 progenitors
- 26 telencephalon (pallium), glutamatergic
- 27 retina (RGC)
- 28 dorsal habenula
- 29 dorsal diencephalon (thalamus)
- 30 radial glia
- 31 retina (cones)
- 32 mid-hind boundary (gabaergic)
- 33 cranial ganglion (vagal)
- 34 hypothalamus
- 35 hypothalamus
- 36 progenitors/differentiating granule cells (hindbrain)
- 37 hypothalamus
- 38 progenitors/neurons (differentiating)
- 39 granule cells
- 40 epidermis (progenitors)
- 41 erythrocytes
- 42 lens
- 43 lens
- 44 eye (cornea), cartilage
- 45 retina (muller glia)
- 46 otic/lateral line
- 47 dorsal habenula
- 48 telencephalon
- 49 neurons
- 50 epithelium (cornea, progenitors)
- 51 cornea
- 52 ventral forebrain (dienc, hyp, poa; gabaergic)
- 53 neurons (dienc, glutamatergic, ZLI)
- 54 ventral habenula
- 55 neurons (telencephalon)
- 56 purkinje neurons (gabaergic ++)
- 57 retina (photoreceptor precursor cells)
- 58 microglia
- 59 neurons (midbrain?, glutamatergic)
- 60 progenitors (cycling)
- 61 hypothalamus
- 62 hypothalamus
- 63 pharangeal arch/pectoral fin (mesoderm)
- 64 retina neurons (horizontal?)
- 65 cartilage
- 66 eye (cornea, epithelium)
- 67 rostral blood island (myeloid)
- 68 oligodendrocytes
- 69 neurons (ganglion)
- 70 neutrophils
- 71 muscle
- 72 retina (rods)
- 73 pigment cell (iridophore)
- 74 glial progenitors
- 75 cranial ganglion (vagal)
- 76 neurons (glutamatergic, midbrain)
- 77 cranial ganglion
- 78 hypothalamus
- 79 retina (RPE)
- 80 olfactory bulb
- 81 eye (non retina)
- 82 neurons (gabaergic)
- 83 oligodendrocytes
- 84 glia (eye)
- 85 glia (mature)
- 86 retina (RPE differentiation)
- 87 dorsal dienchephalon (thalamus)
- 88 otic vesicle
- 89 neural crest derived (pigment/xanthophore)
- 90 rostral blood island (myeloid)
- 91 dermal bone
- 92 dorsal telencephalon (glutamatergic)
- 93 OPC
- 94 (not assigned)

Supplementary Figure 9. Expression of CA receptors in 5dpf brain cells

scRNAseq data from dissected brains and eyes from 5 dpf larval zebrafish published by Raj et al. (2020)[17] were analyzed using the Seurat package (v4.3.0) (https://satijalab.org/seurat/reference/featureplot)[18]. Average expression levels represent log-transformed, library size normalized count per cell as output of the Seurat package. The analysis is based on 31,659 cells dissociated from dissected zebrafish barins at 5 dpf[19]. **(A)** Color coded identified clusters from the publication, for annotation see (E). **(B-D)** Relative expression levels of CA receptors in single cells. **(B)** Expression of DA receptors, combined as indicated. **(C)** Beta2 and beta3-adrenergic receptors which have linked to noradrenaline signaling, combined as indicated. **(D)** Alpha- and beta-adrenergic receptors, combined as indicated. **(E)** Cluster Annotation was retained from original data.

Supplementary Figure 10.

Uncropped images of gel electrophoresis images from Figures 1 and 2

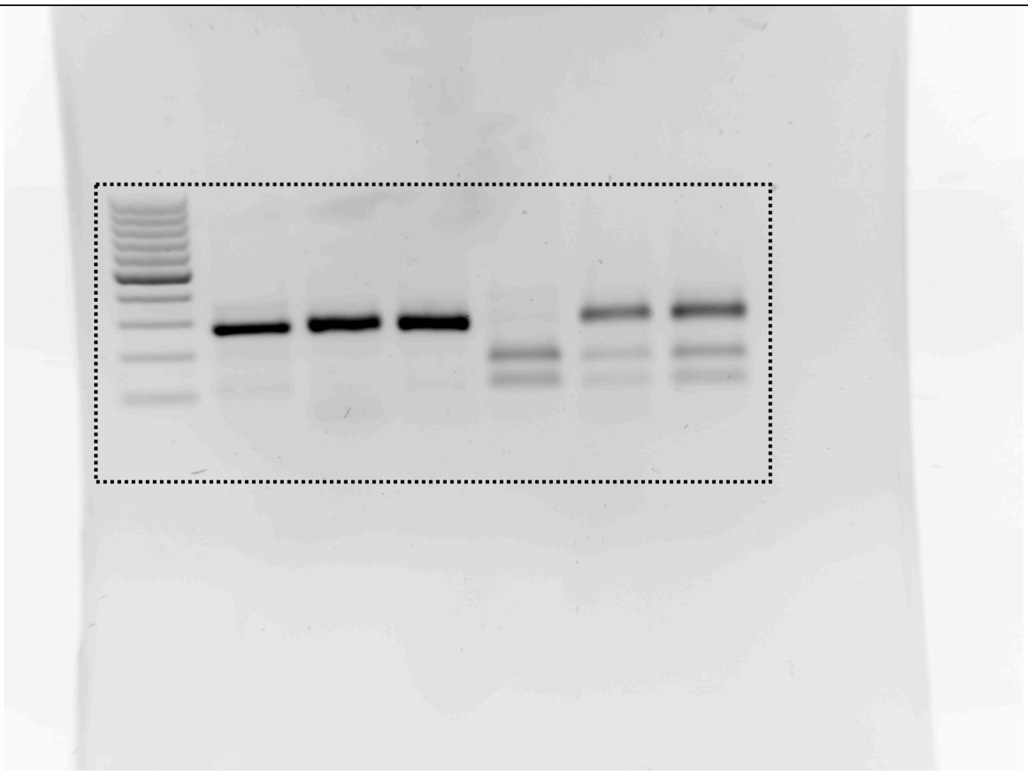	**Uncropped image of gel shown in Figure 1B.**<br><br>Original UV transilluminator image was subjected to grey scale inversion and linear levels adjustment (Photoshop software).
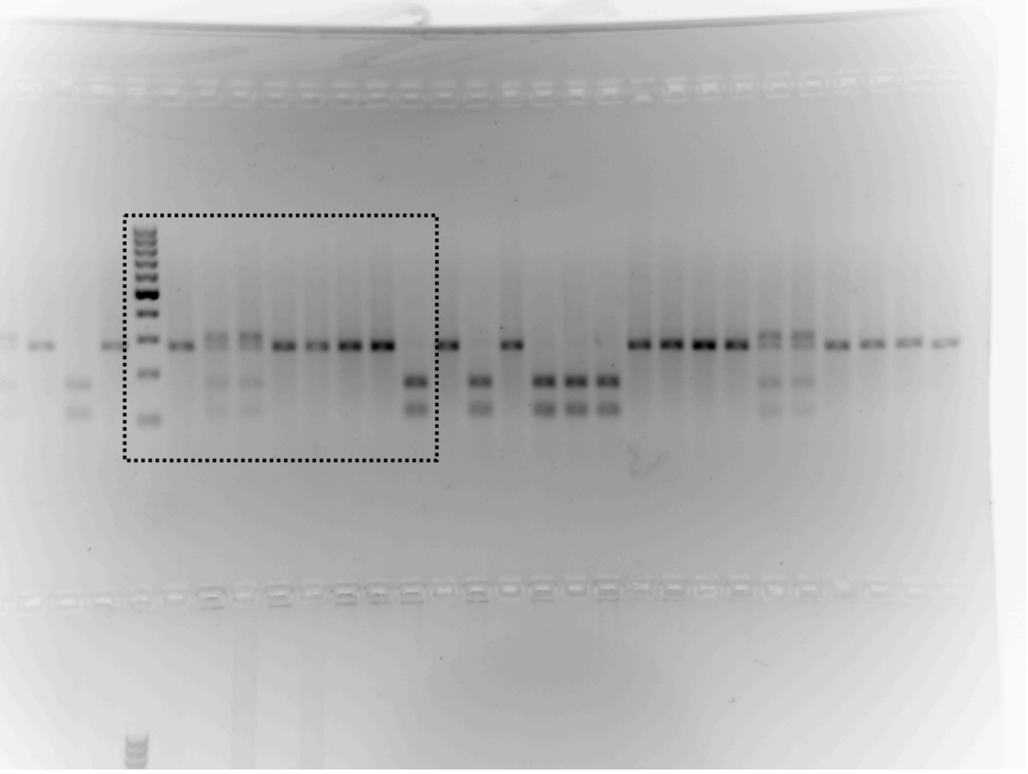	**Uncropped image of gel shown in Figure 1D.**<br><br>Figure 1D shows the 8 lanes to the right of the 100 bp ladder marker (lane 5). The other lanes not included in Figure 1D show genotyping of additional independent samples.<br><br>Original UV transilluminator image was subjected to grey scale inversion and linear levels adjustment (Photoshop software).

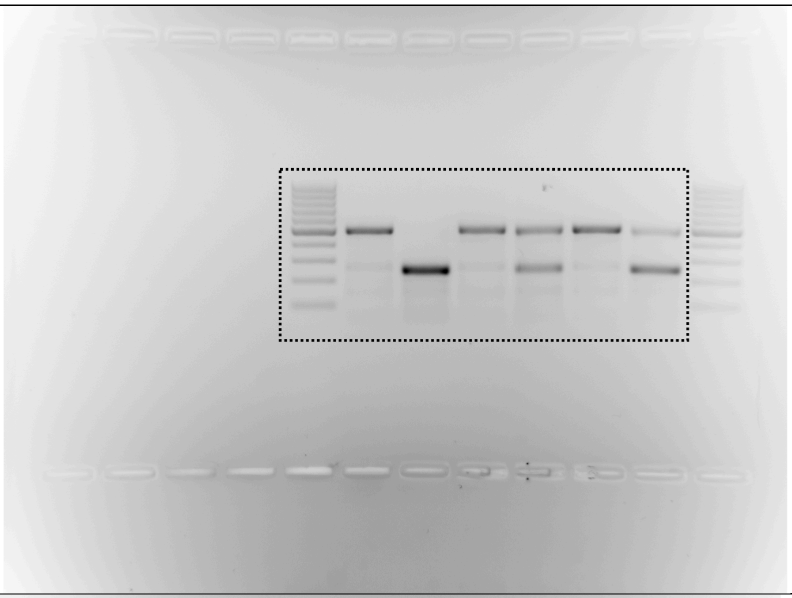	**Uncropped image of gel shown in Figure 2C *th2* exon 1.**<br><br>Original UV transilluminator image was subjected to grey scale inversion and linear levels adjustment (Photoshop software).<br><br>Figure 2C does not contain the rightmost lane with the 100 bp ladder marker (lane 8).
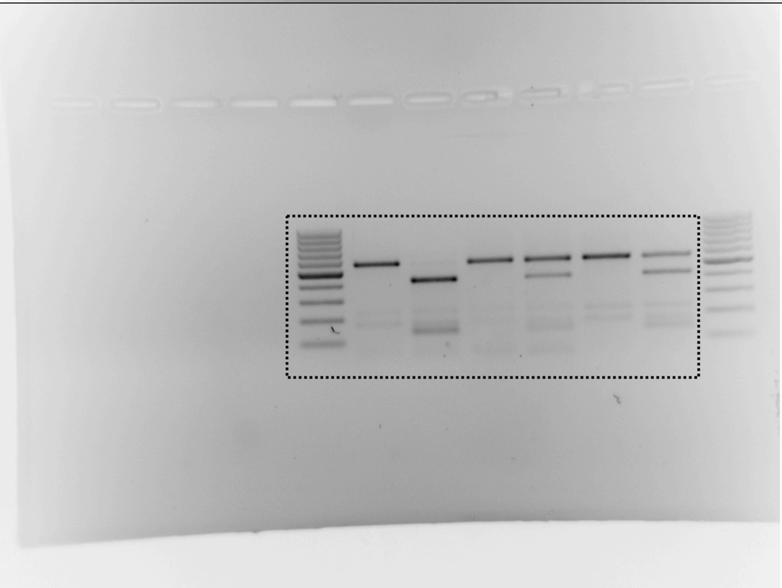	**Uncropped image of gel shown in Figure 2C *th2* exon 12.**<br><br>Original UV transilluminator image was subjected to grey scale inversion and linear levels adjustment (Photoshop software).<br><br>Figure 2C does not contain the rightmost lane with the 100 bp ladder marker (lane 8).
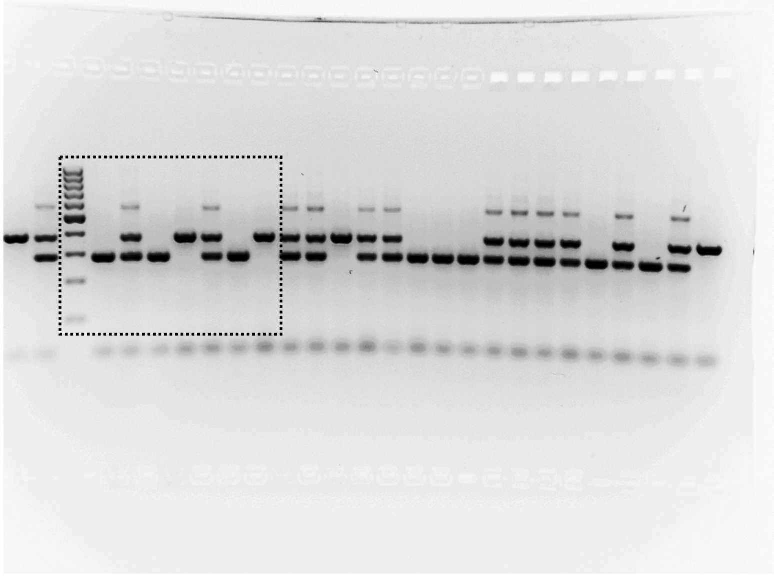	**Uncropped image of gel shown in Figure 2D.**<br><br>Figure 2D shows the 7 lanes to the right of the 100 bp ladder marker (lane 3 from left). The other lanes not included in Figure 2D show genotyping of additional independent samples.<br><br>Original UV transilluminator image was subjected to grey scale inversion and linear levels adjustment (Photoshop software).

Supplementary Table 1: Dopamine ELISA results for WT and *tyr* mutants (related to Figure 3A)

Dopamine ELISA measurements of wild type and *tyr* samples collected from bulked and singles heads, genotypes as indicated.

Collection: heads in bulk (2018)								
Sample	pg/50 heads in 500uL			mean	fold-change relative to AB/TL bulk			mean
WT AB/TL bulk	1452,499	1034,332	1156,975	1214,601722	1,195864	0,851581	0,952554486	0,999999771
WT AB/TL 1/2	567,3588	590,6427	603,605	587,2021623	0,467115	0,486285	0,496957017	0,483452326
WT AB/TL 1/4	168,0637	237,4547	171,3428	192,287057	0,138369	0,1955	0,141069078	0,158312811
WT AB/TL 20	4,821061	25,24798	27,64525	19,23809661	0,003969	0,020787	0,02276075	0,015839013
tyr	543,8847	327,2675	417,2562	429,4694483	0,447788	0,269444	0,343533289	0,353588623
tyr 1/2	250,6668	134,3649	200,5251	195,1856107	0,206378	0,110625	0,165095344	0,160699234

Collection: single heads (2025)								
Sample	pg/50 heads in 500uL			mean	fold-change relative to AB/TL bulk			mean
WT AB/TL heads	3850,65	2699,91	1908,997	2819,852645	1,365551	0,957467	0,67699	1,000000938
WT AB/TL 1/2	2341,82	1133,94		1737,87862	0,830477	0,402126		0,616301796
WT AB/TL 1/4	809,90	666,24		738,0693566	0,287213	0,236268		0,261740645
WT AB/TL 20	62,89305		165,2406	114,0668225	0,022304	0,058599		0,040451379

Supplementary Table 2: Dopamine ELISA for WT and *tyr* mutants statistical analysis (related to Figure 3A)

Dopamine ELISA measurements of wild type and *tyr* samples collected from bulked and singles heads, genotypes as indicated. Statistical analyses (ANOVA, Tukey's HSD Test).

Two-way ANOVA (Type II)

Effect	DFn	DFd	F	p	p<.05	ges
Dilution	5	17	25,325	0,000000256	*	0,882
Collection	1	17	0,788	0,387		0,044
Dilution:Collection	3	17	0,189	0,903		0,032

Two-way factorial ANOVA testing the main effects of Dilution and Collection, and their interaction (Dilution:Collection).

Post-hoc pairwise comparisons

Dilution	.y.	group1	group2	n1	n2	statistic	df	p	p.adj	p.adj.signif	y.position	groups	x	xmin	xmax
WT AB/TL 1/2	Value	bulk	single	3	3	-0,01476	3,00616278	0,989	1	ns	1,3904	bulk, single	1	0,8	1,2
WT AB/TL 1/2	Value	bulk	single	3	2	-0,5964	1,003366593	0,657	1	ns	1,3904	bulk, single	2	1,8	2,2
WT AB/TL 1/4	Value	bulk	single	3	2	-3,27965	2,232403718	0,0704	0,282	ns	1,3904	bulk, single	3	2,8	3,2
WT AB/TL 20	Value	bulk	single	3	2	-1,26491	1,226993865	0,395	1	ns	1,3904	bulk, single	4	3,8	4,2

Post-hoc pairwise comparisons using Welch's two-sample t-tests (comparing bulk vs heads within each Condition), with Bonferroni correction for multiple testing.

Summary Statistics

Condition	mean	sd	n
AB/TL	1,001667	0,247339	6
AB/TL 1/2	0,538	0,167839	5
AB/TL 1/4	0,202	0,064962	5
AB/TL 20	0,024	0,021909	5
tyr	0,353333	0,090738	3
tyr 1/2	0,163333	0,050332	3

One-way ANOVA

Effect	DFn	DFd	F	p	p<.05	ges
Condition	5	21	30,498	5,97E-09	*	0,879

Tukey HSD

term	group1	group2	null.value	estimate	conf.low	conf.high	p.adj	p.adj.signif
Condition	AB/TL	AB/TL 1/2	0	-0,463666667	-0,74371	-0,18363	0,000494	***
Condition	AB/TL	AB/TL 1/4	0	-0,799666667	-1,07971	-0,51963	0,000000185	****
Condition	AB/TL	AB/TL 20	0	-0,977666667	-1,25771	-0,69763	5,65E-09	****
Condition	AB/TL	*tyr*	0	-0,648333333	-0,97535	-0,32132	0,0000492	****
Condition	AB/TL	*tyr 1/2*	0	-0,838333333	-1,16535	-0,51132	0,00000108	****
Condition	AB/TL 1/2	AB/TL 1/4	0	-0,336	-0,62849	-0,04351	0,0184	*
Condition	AB/TL 1/2	AB/TL 20	0	-0,514	-0,80649	-0,22151	0,000239	***
Condition	AB/TL 1/2	*tyr*	0	-0,184666667	-0,52241	0,153073	0,54	ns
Condition	AB/TL 1/2	*tyr 1/2*	0	-0,374666667	-0,71241	-0,03693	0,0241	*
Condition	AB/TL 1/4	AB/TL 20	0	-0,178	-0,47049	0,114491	0,427	ns
Condition	AB/TL 1/4	*tyr*	0	0,151333333	-0,18641	0,489073	0,726	ns
Condition	AB/TL 1/4	*tyr 1/2*	0	-0,038666667	-0,37641	0,299073	0,999	ns
Condition	AB/TL 20	*tyr*	0	0,329333333	-0,00841	0,667073	0,0587	ns
Condition	AB/TL 20	*tyr 1/2*	0	0,139333333	-0,19841	0,477073	0,787	ns
Condition	*tyr*	*tyr 1/2*	0	-0,19	-0,5676	0,187604	0,623	ns

Supplementary Table 3: *th* cell counts (related to Figures 4 and S3)

Cell counts and statistical analyses of *th* expressing catecholaminergic neurons for each analysed cell cluster, genotypes as indicated. For abbreviations of anatomical cluster names see Figure 4 legend.

		**Number of *th*+ cells in DA Clusters**											
#	Genotype	DC1	DC2	DC3	DC4	DC5	DC6	DC7	LC	PO	SP	OB	Pr
1	AB/TL	27	2	1	6	8	8	6	6	8	11	11	32
2	AB/TL	27	4	1	5	8	9	8	5	8	13	15	29
3	AB/TL	35	4	1	7	6	9	5	7	7	12	16	28
4	AB/TL	34	4	1	7	8	8	6	9	9	10	12	30
5	*mitfa-/-*	29	3	2	3	8	13	10	11	8	14	20	31
6	*mitfa-/-*	33	3	3	3	10	8	9	7	7	13	18	27
7	*mitfa-/-*	48	5	4	6	5	23	5	10	12	13	22	28
8	*mitfa-/-*	46	7	3	7	9	23	6	8	10	13	25	29
9	*mitfa-/-*	39	3	2	2	14	12	9	8	7	13	22	34
10	*mitfa-/-*	36	2	2	3	16	12	11	6	7	9	23	29
11	*th-/-, tyr-/-, mitfa-/-*	39	3	1	3	11	20	9	8	7	9	24	25
12	*th-/-, tyr-/-, mitfa-/-*	37	5	2	4	10	17	13	7	9	12	19	27
13	*th-/-, tyr-/-, mitfa-/-*	27	3	1	2	7	14	7	4	8	10	20	28
14	*th-/-, tyr-/-, mitfa-/-*	31	5	2	4	9	15	6	4	7	8	21	35
15	*th-/-, tyr-/-, mitfa-/-*	29	5	2	3	5	16	10	6	8	9	9	34
16	*th-/-, tyr-/-, mitfa-/-*	27	3	3	3	6	14	9	7	11	12	9	32
17	*th2-/-, tyr-/-, mitfa-/-*	20	6	2	7	11	12	7	8	6	10	22	30
18	*th2-/-, tyr-/-, mitfa-/-*	26	5	3	6	9	15	6	6	9	8	17	25
19	*th2-/-, tyr-/-, mitfa-/-*	27	2	3	4	7	17	6	7	6	11	20	31
20	*th2-/-, tyr-/-, mitfa-/-*	29	3	2	4	10	18	9	6	9	13	25	36
21	*th2-/-, tyr-/-, mitfa-/-*	31	2	2	4	10	24	10	6	7	12	21	21
22	*th2-/-, tyr-/-, mitfa-/-*	29	2	3	4	10	21	11	5	8	13	20	26
23	*th-/-, th2-/-, tyr-/-, mitfa-/-*	27	3	1	3	9	14	12	6	5	11	23	31
24	*th-/-, th2-/-, tyr-/-, mitfa-/-*	28	3	2	5	5	13	9	3	11	10	20	31
25	*th-/-, th2-/-, tyr-/-, mitfa-/-*	28	5	1	4	6	16	8	5	7	9	19	31
26	*th-/-, th2-/-, tyr-/-, mitfa-/-*	25	5	2	6	8	14	7	5	7	8	20	34
27	*th-/-, th2-/-, tyr-/-, mitfa-/-*	32	4	1	5	11	13	10	6	5	11	18	27
28	*th-/-, th2-/-, tyr-/-, mitfa-/-*	30	3	1	3	11	14	4	7	7	8	16	22

Supplementary Table 4: *th2* cell counts (related to Figures 4 and S3)

Cell counts and statistical analyses of *th2* expressing catecholaminergic neurons for each analysed cell cluster, genotypes as indicated. For abbreviations of anatomical cluster names see Figure 4 legend.

		Number of *th2*+ cells in DA Clusters		
#	Genotype	DC1	DC3	DC7
1	AB/TL	9	5	23
2	AB/TL	7	5	25
3	AB/TL	7	6	29
4	AB/TL	7	7	33
5	*mitfa-/-*	10	6	30
6	*mitfa-/-*	12	5	36
7	*mitfa-/-*	8	4	30
8	*mitfa-/-*	8	4	36
9	*mitfa-/-*	10	6	33
10	*mitfa-/-*	11	6	37
11	*th-/- tyr-/- mitfa-/-*	8	7	37
12	*th-/- tyr-/- mitfa-/-*	7	7	33
13	*th-/- tyr-/- mitfa-/-*	5	7	26
14	*th-/- tyr-/- mitfa-/-*	5	6	20
15	*th-/- tyr-/- mitfa-/-*	3	6	35
16	*th-/- tyr-/- mitfa-/-*	3	5	34

Supplementary Table 5: Cell counts of *mnx1a:GFP* positive cells at 33 hpf (related to Figure 6B)
Cell counts and statistical analysis for the data shown in Figure 6B 33 hpf.

		Cell countings in Tg(mnx1a:GFP) at 33 hpf									
		Somite 7°		Somite 8°		Somite 9°		Somite 10°		Total number of primary neurons	
#	Genotype	Right	Left	Right	Left	Right	Left	Right	Left	per larva	per somite
1	ABTL	14	14	16	17	14	19	13	15	122	30,5
2	ABTL	11	22	13	15	22	22	21	22	148	37,0
3	ABTL	10	13	16	14	10	15	8	18	104	26,0
4	ABTL	20	19	22	24	23	25	21	29	183	45,8
5	ABTL	21	18	21	22	20	28	23	23	176	44,0
1	*tyr-/-*	8	11	10	13	12	12	13	13	92	23,0
2	*tyr-/-*	12	14	14	18	13	19	14	16	120	30,0
3	*tyr-/-*	25	26	26	24	26	27	29	29	212	53,0
4	*tyr-/-*	23	24	26	25	20	24	26	30	198	49,5
5	*tyr-/-*	24	22	21	22	25	24	29	23	190	47,5
1	*th-/-, th2-/-, tyr-/-*	22	25	21	18	14	17	19	23	159	39,8
2	*th-/-, th2-/-, tyr-/-*	17	19	17	20	15	16	20	22	146	36,5
3	*th-/-, th2-/-, tyr-/-*	18	18	18	22	22	22	21	23	164	41,0
4	*th-/-, th2-/-, tyr-/-*	15	20	24	22	20	22	20	22	165	41,3
5	*th-/-, th2-/-, tyr-/-*	18	19	23	21	23	21	23	25	173	43,3

One-way ANOVA				
F Welch (2, 33.3) = 1.37, p = 0.27, effect size (ω2) = 0.02, CI95%[0.00, 1]				
ABTL	*tyr*	*th, th2, tyr*	*p-value*	significance
x		x	0,75000	ns
	x	x	1	ns
x	x		0,99	ns
Method: One way analysis of means (not assuming equal variances)				
F Welch (a, b)				
F : Confidence distribution				
a = degrees of freedom				
b = degrees of freedom error				
Welch's ANOVA with Games-Howell pairwise comparisons and Holm p adjustment method				

Supplementary Table 6: Cell counts of *mnx1a:GFP* positive cells at 3 dpf (related to Figure 6B)

Cell counts and statistical analysis for the data shown in Figure 6B 3 dpf.

		Cell countings in Tg(mnx1a:GFP) at 3 dpf				
		Somite 7°	Somite 8°	Somite 9°	Total number of secondary neurons	
#	Genotype				per larva	per somite
1	ABTL	33	34	33	100	33,3
2	ABTL	34	40	32	106	35,3
3	ABTL	33	39	32	104	34,7
4	ABTL	40	30	35	105	35,0
5	ABTL	36	35	33	104	34,7
1	*tyr-/-*	36	36	46	118	39,3
2	*tyr-/-*	31	24	32	87	29,0
3	*tyr-/-*	33	32	33	98	32,7
4	*tyr-/-*	38	32	31	101	33,7
5	*tyr-/-*	38	42	41	121	40,3
1	*th-/-, th2-/-, tyr-/-*	28	33	27	88	29,3
2	*th-/-, th2-/-, tyr-/-*	33	32	37	102	34,0
3	*th-/-, th2-/-, tyr-/-*	43	30	45	118	39,3
4	*th-/-, th2-/-, tyr-/-*	23	32	26	81	27,0
5	*th-/-, th2-/-, tyr-/-*	42	35	38	115	38,3

One-way ANOVA				
F Welch (2, 24.99) = 0.21, p = 0.81, effect size (ω2) = 0.0, CI95%[0.00, 1]				
ABTL	*tyr*	*th, th2, tyr*	*p-value*	significance
x		x	1	ns
	x	x	1	ns
x	x		1	ns
Method: One way analysis of means (not assuming equal variances)				
F Welch (a, b)				
F : Confidence distribution				
a = degrees of freedom				
b = degrees of freedom error				
Welch's ANOVA with Games-Howell pairwise comparisons and Holm p adjustment method				

Supplementary Table 7: HGC phenotypes 3 dpf (related to Figure 7B)

Embryos from a *th+/-, th2+/-, tyr-/-* cross were analysed at 3 dpf for HGC phenotype. 180 embryos with HGC[+] phenotype were identified. 189 HGC[-] embryos were randomly selected from the cross as HGC[-] population. Genotypes for each embryo were determined by PCR after sorting.

		Counts and percentage of larvae with (HGC+) or without (HGC-) retention of HGC at 3dpf			
		**Mutant alleles**	**Counts**	**Total counts / genotype**	**% / phenotype**
HGC retention	HGC[+]	***tyr***	41	170	22,8
		th2, tyr	16	64	8,9
		th, tyr	89	98	49,4
		th, th2, tyr	34	37	18,9
		**Total counts / phenotype**	180		
	HGC[-]	***tyr***	129	170	68,3
		th2, tyr	48	64	25,4
		th, tyr	9	98	4,8
		th, th2, tyr	3	37	1,6
		**Total counts / phenotype**	189		

Supplementary Table 8: Levels of HGC retention among genotypes from 3 dpf to 5 dpf (related to Figure 7E)

A random subset of 92 embryos classified as HGC+ retention phenotype at 3 dpf (Figure 7B and Supplementary Table 7) were reanalysed at 5 dpf. The percentages were extrapolated based on the analysis group of 92 larvae with the 3 dpf HGC retention phenotype, and not on the total cross (see legend Table 5).

		Counts and percentage of larvae with (HGC+) or without (HGC-) retention of HGC at 3dpf				
		**Mutant alleles**	**Counts**	**Total counts / genotype**	**% / genotype**	
**HGC retention**	**HGC[+]**	***tyr***	41	170	24,1	
		th2, tyr	16	64	25,0	
		th, tyr	89	98	90,8	
		th, th2, tyr	34	37	91,9	
		**Total counts / phenotype**	180			
	**HGC[-]**	***tyr***	129	170	75,9	
		th2, tyr	48	64	75,0	
		th, tyr	9	98	9,2	
		th, th2, tyr	3	37	8,1	
		**Total counts / phenotype**	189			

		Counts and percentage of larvae carrying "high", "low "and "none" HGC retention at 5dpf from a subset of HGC+ at 3dpf				Extrapolated values of HGC+ genotypes at 5dpf from the total pool of 3dpf larvae
		**Mutant alleles**	**Counts**	**Total counts / genotype**	**% / genotype**	**% genotypes / total**
**HGC Phenotype at 5 dpf**	**High**	***tyr***	0	33	0,0	0,0
		th2, tyr	0	11	0,0	0,0
		th, tyr	18	30	60,0	54,5
		th, th2, tyr	13	18	72,2	66,4
		**Total counts/phenotype**	31			
	**Low**	***tyr***	9	33	27,3	6,6
		th2, tyr	5	11	45,5	11,4
		th, tyr	8	30	26,7	24,2
		th, th2, tyr	4	18	22,2	20,4
		**Total counts/phenotype**	26			
	**None**	***tyr***	24	33	72,7	93,4
		th2, tyr	6	11	54,5	88,6
		th, tyr	4	30	13,3	21,3
		th, th2, tyr	1	18	5,6	13,2
		**Total counts/phenotype**	35,0			
		**Total**	92,0			

Supplementary Table 9: HR and HRV temperature dependence data (related to Figure 8)

HR and HRV measurements for the analysis shown in Figure 8 and in Supplementary Figure 6. Outliers are shown in red, but the full data set is shown in Figure 8 without outliers removed. Outlier exclusion would have not affected the significance of the results reported in the main Figure 8.

		Heart Rate (bpm)					Heart Rate Variability (RMSSD)				
#	Mutant alleles	36°C	32°C	28°C	24°C	20°C	36°C	32°C	28°C	24°C	20°C
1	*tyr*	286	280	186	156	106	107,7215	66,09759	32,82016	85,07611	139,3167
2	*tyr*	319	285	200	152	100	88,58375	83,87828	35,27382	62,51918	189,336
3	*tyr*	293	273	182	144	90	61,89187	64,74135	30,72133	57,42937	100,7348
4	*tyr*	330	296	204	144	98	46,88003	52,6908	46,74306	61,36416	75,01
5	*tyr*	322	260	188	162	90	68,68655	77,43134	29,63758	107,13	140,0555
6	*tyr*	297	252	196	154	94	93,67247	79,96436	31,53104	45,14683	91,6163
7	*tyr*	308	300	182	122	106	56,05314	69,00248	58,09609	63,91861	59,34547
8	*tyr*	285	265	192	120	116	83,16022	77,49286	31,24652	76,17694	56,68024
9	*tyr*	328	279	194	166	96	64,81804	83,80636	32,90577	133,217	90,3101
10	*tyr*	338	265	182	154	98	75,60308	75,29701	34,18196	50,41949	96,32854
11	*tyr*	326	262	196	138	114	60,1674	63,87682	36,48287	152,2159	73,21775
12	*tyr*	308	267	216	124	92	90,24408	63,30177	35,07694	155,7793	151,999
13	*tyr*	307	246	194	158	102	80,22176	83,49321	32,33305	135,334	67,46866
14	*tyr*	320		194	150	96	61,55511		32,18782	87,12507	95,38563
15	*tyr*	315		180	162	93	73,27937		45,20831	48,2515	92,7529
16	*tyr*	300		176	168	92	72,60828		35,6943	69,11927	114,1035
17	*tyr*	325		183	160		63,07397		34,87191	42,2983	
18	*tyr*			196	164				32,14481	51,013	
19	*tyr*				144					61,46183	
20	*tyr*				146					51,22212	
1	*th2, tyr*	308	291	198	154	98	93,17958	62,65947	26,45886	105,4713	123,2268
2	*th2, tyr*	296	246	186	132	104	71,59201	86,64761	31,90577	45,80739	85,72436
3	*th2, tyr*	324	232	190	168	104	67,63868	61,71587	30,06058	112,6212	77,08424
4	*th2, tyr*	306	262	184	140	102	62,45868	63,77705	33	76,6119	63,45077
5	*th2, tyr*	323	257	206	148	112	73,24536	60,99594	30,82541	37,57733	79,03946
6	*th2, tyr*	334	271	190	134	104	60,46547	68,91261	34,54368	64,81578	166,7278
7	*th2, tyr*	308	240	210		102	53,54466	67,45017	31,06445		162,3385
8	*th2, tyr*			198		92			27,4142		74,11747
9	*th2, tyr*			178					38,5357		
10	*th2, tyr*			178					38,5357		
11	*th2, tyr*			180					36,01404		
12	*th2, tyr*			178					28,36151		
13	*th2, tyr*			182					30,12474		
14	*th2, tyr*			176					23,20003		
1	*th, tyr*	288	271	202	143	108	123,0221	76,66789	35,67324	48,65021	61,55332
2	*th, tyr*	297	263	190	147	100	57,1492	68,06733	29,86726	53,71923	71,34009
3	*th, tyr*	294	290	204	153	102	97,78232	60,59686	29,48771	74,62595	54,28357
4	*th, tyr*	306	267	198	161	112	64,16939	112,0124	37,45274	58,32737	60,24948
5	*th, tyr*	271	262	180	157	114	66,82537	91,35517	32	44,09452	46,87693
6	*th, tyr*	340	274	204	143	112	56,51163	70,92915	35,57103	65,20056	94,54244
7	*th, tyr*	278	273	190	149	90	80,91331	96,3509	40,87615	43,44923	78,02884
8	*th, tyr*	311	272	202	141	102	67,98951	61,67954	23,10195	44,9384	71,9975
9	*th, tyr*	270	273	162	167	100	96,6092	75,29038	30,96909	47,19592	71,34009
10	*th, tyr*	294	246		121	100	75,01253	108,3021		65,30412	55,38032
11	*th, tyr*	273	255		123	92	73,86858	70,24675		51,7541	53,43781
12	*th, tyr*	256	258		143	96	72,90923	75,13563		68,755	121,5232
13	*th, tyr*	293	274		149	100	71,59201	99,25358		50,41117	106,2546
14	*th, tyr*	292			137	105	76,85113			49,96952	71,81041
15	*th, tyr*	300			145	104	66,16516			46,53777	81,7525
16	*th, tyr*	288			163	96	67,10259			71,3109	95,30332
17	*th, tyr*				155					43,76922	
18	*th, tyr*				167					58,95557	
19	*th, tyr*				149					54,91532	
20	*th, tyr*				169					41,35717	
21	*th, tyr*				137					45,4644	
22	*th, tyr*				157					75,30541	
23	*th, tyr*				155					51,70333	
24	*th, tyr*				147					72,60334	
25	*th, tyr*				149					39,27255	
26	*th, tyr*				141					48,65903	
27	*th, tyr*				131					59,18615	
1	*th, th2, tyr*	283	260	188	146	99	83,23306	74,95233	36,80755	54,9704	81,25308
2	*th, th2, tyr*	296	240	190	158	104	63,61967	72,19495	31,00909	46,47334	58,26764
3	*th, th2, tyr*	296	267	156	116	96	67,48498	83,14118	26,97367	50,31093	69,83428
4	*th, th2, tyr*	327	290	176	142	98	49,23563	72,87555	28,74262	46,38196	75,31185
5	*th, th2, tyr*	302	286		132	96,6	77,84276	57,94391		38,4067	59,03219
6	*th, th2, tyr*	282	269		148	92	60,18815	78,61215		38,98576	60,84899
7	*th, th2, tyr*		260		140	90		74,9574		44,60666	66,29017
8	*th, th2, tyr*				118					45,56863	
9	*th, th2, tyr*				150					46,54871	
10	*th, th2, tyr*				172					46,16255	
	Red: Outlier										

Supplementary Table 10: HR and HRV temperature dependence statistical analysis - summary (related to Figure 8)

Two-way ANOVA and Tukey's multiple comparison results for HR and HRV related to Figure 8 and Supplementary Figure 6 (details in Supplementary Data 6 and 7).

**Two-way ANOVA Heart Rate (Type III)**					
	Sum Sq	Df	F value	Pr(>F)	
(Intercept)	7649653,079	1	42566,95009	1,06E-254	***
Temperature	1097495,039	4	1526,769125	1,11E-159	***
Genotype	1789,989716	3	3,320168122	0,02067791	*
Temperature:Genotype	5451,627389	12	2,527992104	0,003860454	**
Residuals	39715,63213	221	NA	NA	
Signif. codes: 0 '***' 0.001 '**' 0.01 '*' 0.05 '.' 0.1 ' ' 1					

**Two-way ANOVA Heart Rate Variability (Type III)**					
	Sum Sq	Df	F value	Pr(>F)	
(Intercept)	819858,5678	1	2017,400815	4,35E-113	***
Temperature	57656,19767	4	35,468209	7,05E-23	***
Genotype	6700,097925	3	5,495575108	0,001168529	**
Temperature:Genotype	14396,28825	12	2,952042053	0,000783499	***
Residuals	89812,96239	221	NA	NA	
Signif. codes: 0 '***' 0.001 '**' 0.01 '*' 0.05 '.' 0.1 ' ' 1					

Supplementary Table 11: Locomotor activity in ABTL and *tyr* mutants related to Supplementary Figure 7)

Spontaneous locomotor activity for control genotypes ABTL and *tyr-/-* shown in Supplementary Figure 7.

	Distance moved (m) during 1h at 5 dpf	
#	***ABTL***	***tyr-/-***
1	6,24466	6,69109
2	7,16003	10,83680
3	6,81733	12,23210
4	13,103	5,19823
5	4,2769	6,96033
6	10,6337	6,08805
7	4,96107	6,70627
8	4,35283	5,10805
9	9,227	4,19258
10	4,36307	6,32804
11	4,91063	11,54040
12	9,91557	12,55600
13	4,34285	5,17670

Supplementary Table 12: Locomotor activity data (related to Figure 9A)

Spontaneous locomotor activity measurements of larvae obtained from *th+/-, th2+/-, tyr-/-* in-crosses. Analysis is shown in Figure 9A. Figure 9A shows the full data set, without outliers removed. Outlier exclusion does not affect the significance of the results reported in the main Figure 9A.

	Distance moved (m) during 1h at 5 dpf			
#	*tyr-/-*	*th2-/-, tyr-/-*	*th-/-, tyr-/-*	*th-/-, th2-/-, tyr-/-*
1	0,941833	3,791847	0,578892	0,748789
2	1,824384	1,539639	0,891090	0,350019
3	0,185017	5,144964	1,008507	1,242789
4	7,352480	2,887850	4,165408	0,471955
5	0,031629	2,139605	**8,7644037***	0,064969
6	5,064168	4,542031	0,508746	0,483108
7	1,682724	7,934385	**8,17235404***	3,936040
8	6,379557	10,320869	0,565751	1,726496
9	5,251691	6,224856	0,682749	1,496512
10	1,538581	1,597394	3,980344	0,641845
11	4,542031	1,629150	0,027472	3,124929
12	2,588609	6,583556	0,770117	
13	6,865873	0,087526	0,244321	
14	1,485732	4,560621	0,118159	
15	7,934385	6,917518	0,001826	
16	4,899715	5,493605	0,822271	
17	6,888445	0,167223	0,624406	
18	1,597394		1,349047	
19	6,583556		0,010864	
20	6,718518		3,192713	
21	1,503058		0,113378	
22	1,582165		0,208252	
23	6,917518		1,620762	
24			4,120440	
25			2,106841	
26			0,224676	
27			0,444392	
28			2,830125	
29			0,024415	
30			0,857431	
31			1,652705	

***: Outliers**

($\chi 2$ Kruskal-Wallis (3) = 19.95, p = 0.000174, effect size (ε^2 (rank)) = 0.25, CI 95% [0.12, 1.00])

χ2: chi-square approximation

Supplementary Table 13: Optomotor response data (related to Figure 9C)

Proportion of responders and non-responders among genotypes

Response		Counts and percentage of larval responses to moving gratings at 5 dpf			
		Mutant alleles	Counts	Total counts / genotype	% / genotype
Response	OMR	*tyr*	49	60	81,7
		th2, tyr	39	44	88,6
		th, tyr	55	78	70,5
		th, th2, tyr	36	40	90,0
		Total counts / phenotype	179		
	No OMR	*tyr*	1	60	1,7
		th2, tyr	3	44	6,8
		th, tyr	10	78	12,8
		th, th2, tyr	2	40	5,0
		Total counts / phenotype	16		
	No Movemer	*tyr*	10	60	16,7
		th2, tyr	2	44	4,5
		th, tyr	13	78	16,7
		th, th2, tyr	2	40	5,0
		Total counts / phenotype	27		

Supplementary Table 14: Optomotor response statistical tests (related to Figure 10)

Genotype distributions were compared using the two-sample Kolmogorov-Smirnov test. Pairs of compared genotypes are shown with an "x". D: Maximum distance between the empirical distribution functions of two samples. Results of Kruskal-Wallis with Dunn's pairwise comparisons and Holm *p* adjustment method for each kinematic parameter analyzed considering the outliers (Outliers Present) and not considering them (Outliers Removed).

**Kruskal-Wallis one-way ANOVA without outlier analysis**					
Kinematic Parameter	Test	*p*	significance	effect size	Confidence Intervals (95%)
Latency	χ^2 Kruskal-Wallis (3) = 14.02	0,00288	**	ε^2 = 0.06	[0.03, 1.00]
Directionality	χ^2 Kruskal-Wallis (3) = 7.73	0,05	ns	ε^2 = 0.03	[0.01, 1.00]
Velocity	χ^2 Kruskal-Wallis (3) = 18.26	0,00039	***	ε^2 = 0.08	[0.03, 1.00]
Speed	χ^2 Kruskal-Wallis (3) = 17.50	0,000557	***	ε^2 = 0.07	[0.04, 1.00]
χ^2: chi-squared approximation					

**Kruskal-Wallis one-way ANOVA (Outliers removed)**					
Kinematic Parameter	Test	*p*	significance	effect size	Confidence Intervals (95%)
Latency	χ^2 Kruskal-Wallis (3) = 25.33	0,00001	****	(ε^2) = 0.12	[0.05, 1.00]
Directionality	χ^2 Kruskal-Wallis (3) = 15.78	0,00126	**	(ε^2) = 0.07	[0.04, 1.00]
Velocity	χ^2 Kruskal-Wallis (3) = 21.13	0,00009	****	(ε^2) = 0.09	[0.04, 1.00]
Speed	χ^2 Kruskal-Wallis (3) = 20.19	0,000155	***	(ε^2) = 0.09	[0.03, 1.00]

	**OMR data without outlier analysis**							**Dunn's pairwise multiple comparisons and Holm p adjustment method**			
	**Comparison of two distributions using the two-sample Kolmogorov-Smirnov test**							**Outliers Present**		**Outliers Removed**	
	tyr	*th2, tyr*	*th, tyr*	*th, th2, tyr*	D	*p-value*	significance	p-value	significance	p-value	significance
**Latency**	x	x			0,08090	0,980500	ns	0,81275	ns	0,89486	ns
	x		x		0,24660	0,014450	*	0,09895	ns	0,00527	**
	x			x	0,33730	0,003110	**	0,01025	*	0,00009	****
		x	x		0,23340	0,108600	ns	0,13058	ns	0,04055	*
		x		x	0,32540	0,025280	*	0,02000	*	0,00180	**
			x	x	0,20401	0,255800	ns	0,49000	ns	0,27448	ns
**Directionality**	x	x			0,14286	0,535400	ns	0,99990	ns	0,99990	ns
	x		x		0,19500	0,090020	*	0,60987	ns	0,02813	*
	x			x	0,27302	0,029010	*	0,43676	ns	0,02813	*
		x	x		0,20765	0,198100	ns	0,87333	ns	0,02813	*
		x		x	0,30952	0,038140	*	0,60967	ns	0,02813	*
			x	x	0,11384	0,888300	ns	0,99990	ns	0,99999	ns
**Velocity**	x	x			0,19524	0,183000	ns	0,49062	ns	0,25809	ns
	x		x		0,33630	0,000210	***	0,02347	*	0,01857	*
	x			x	0,32302	0,005420	**	0,01858	*	0,01720	*
		x	x		0,36222	0,001970	**	0,00703	**	0,00158	**
		x		x	0,36111	0,009250	**	0,00501	**	0,00149	**
			x	x	0,15483	0,581800	ns	0,57270	ns	0,60129	ns
**Speed**	x	x			0,21429	0,112700	ns	0,50396	ns	0,26267	ns
	x		x		0,36643	0,000037	****	0,02581	*	0,02075	*
	x			x	0,34048	0,002819	**	0,02581	*	0,02566	*
		x	x		0,33841	0,004759	**	0,00742	**	0,00163	**
		x		x	0,43254	0,000881	***	0,00781	**	0,00266	**
			x	x	0,14845	0,632300	ns	0,73639	ns	0,78677	ns

Supplementary Table 15: Sequence of oligos used for *th2* HCR in situ detection (related to Methods)

th2_B5_P1	CTCACTCCCAATCTCTATaaCTGTCGCGCCGCGCGTCCTCAATCA
th2_B5_P2	GGGCCCGGAGATGAAGAGCCGCTCTaaCTACCCTACAAATCCAAT
th2_B5_P3	CTCACTCCCAATCTCTATaaCTCAAAGACTTTGCCAGCCTTGAAG
th2_B5_P4	CAGGTGTAGCAGCTTGGTTTCGAAAaaCTACCCTACAAATCCAAT
th2_B5_P5	CTCACTCCCAATCTCTATaaTCTTGCTCTTTCTGCTCGCTCGACT
th2_B5_P6	AGAACTCCAGATCTTCTCCACTGCTaaCTACCCTACAAATCCAAT
th2_B5_P7	CTCACTCCCAATCTCTATaaAGGTCAGAAATCTTCTTTGGAAACC
th2_B5_P8	TTTGTTATCAGATGATTGCATGTATaaCTACCCTACAAATCCAAT
th2_B5_P9	CTCACTCCCAATCTCTATaaTCGGAGCAGTGCACTTCACACCGCA
th2_B5_P10	TTTAAAGAGTTGATGAAGATGTCGGaaCTACCCTACAAATCCAAT
th2_B5_P11	CTCACTCCCAATCTCTATaaATCTGGTCTTCTCCATACAGACGTT
th2_B5_P12	AAAGCAGACACCTCTCTGAGCTGAGaaCTACCCTACAAATCCAAT
th2_B5_P13	CTCACTCCCAATCTCTATaaACCTCCCATGTGGCATTCTCTTCTG
th2_B5_P14	GGGTAAAGGCTCCTCAGAGTCCTGTaaCTACCCTACAAATCCAAT
th2_B5_P15	CTCACTCCCAATCTCTATaaCGTGCTTATAATTGAAAGCCATTTC
th2_B5_P16	TGTATTCCACTACTGGTAAAGGGTCaaCTACCCTACAAATCCAAT
th2_B5_P17	CTCACTCCCAATCTCTATaaACACAAAGTAAACTGGTTGATATGA
th2_B5_P18	TCTGTTTGGCGTCCTCAAAGCTCTCaaCTACCCTACAAATCCAAT
th2_B5_P19	CTCACTCCCAATCTCTATaaAGCTGGGTCGAATGGCTTGTACTGA
th2_B5_P20	ATCTTGGTATGGTTGAACTGCAGTCaaCTACCCTACAAATCCAAT

Supplementary References

1 Eisenhofer, G. *et al.* Tyrosinase: a developmentally specific major determinant of peripheral dopamine. *Faseb J* **17**, 1248-1255 (2003). https://doi.org:10.1096/fj.02-0736com

2 Rink, E. & Wullimann, M. F. The teleostean (zebrafish) dopaminergic system ascending to the subpallium (striatum) is located in the basal diencephalon (posterior tuberculum). *Brain Research* **889**, 316-330 (2001). https://doi.org:Doi 10.1016/S0006-8993(00)03174-7

3 Rink, E. & Wullimann, M. F. Development of the catecholaminergic system in the early zebrafish brain: an immunohistochemical study. *Brain Res Dev Brain Res* **137**, 89-100 (2002). https://doi.org:10.1016/s0165-3806(02)00354-1

4 Yamamoto, K. & Vernier, P. The evolution of dopamine systems in chordates. *Frontiers in Neuroanatomy* **5** (2011). https://doi.org:10.3389/fnana.2011.00021

5 Smeets, W. J. A. J. & Reiner, A. in *Phylogeny and Development of Catecholamine Systems in the CNS of Vertebrates* (eds W.J.A.J. Smeets & A. Reiner) 463-481 (Cambridge University Press, 1994).

6 Schredelseker, T., Veit, F., Dorsky, R. I. & Driever, W. Bsx Is Essential for Differentiation of Multiple Neuromodulatory Cell Populations in the Secondary Prosencephalon. *Front Neurosci* **14**, 525 (2020). https://doi.org:10.3389/fnins.2020.00525

7 Holzschuh, J., Ryu, S., Aberger, F. & Driever, W. Dopamine transporter expression distinguishes dopaminergic neurons from other catecholaminergic neurons in the developing zebrafish embryo. *Mech Dev* **101**, 237-243 (2001). https://doi.org:10.1016/s0925-4773(01)00287-8

8 Armbruster, D., Mueller, T. & Driever, W. Dopaminergic Neurons in the Zebrafish Subpallium Belong to the Extended Medial Amygdala. *J Comp Neurol* **533**, e70079 (2025). https://doi.org:10.1002/cne.70079

9 Reiner, A. & Northcutt, R. G. An Immunohistochemical Study of the Telencephalon of the African Lungfish, Protopterus-Annectens. *J Comp Neurol* **256**, 463-481 (1987). https://doi.org:DOI 10.1002/cne.902560313

10 Altbürger, C., Holzhauser, J. & Driever, W. CRISPR/Cas9-based QF2 knock-in at the tyrosine hydroxylase (th) locus reveals novel th-expressing neuron populations in the zebrafish mid- and hindbrain. *Frontiers in Neuroanatomy* **17** (2023). https://doi.org:10.3389/fnana.2023.1196868

11 Ma, P. M. Catecholaminergic Systems in the Zebrafish .1. Number, Morphology, and Histochemical-Characteristics of Neurons in the Locus-Coeruleus. *J Comp Neurol* **344**, 242-255 (1994). https://doi.org:DOI 10.1002/cne.903440206

12 Ma, P. M. Catecholaminergic systems in the zebrafish .3. Organization and projection pattern of medullary dopaminergic and noradrenergic neurons. *J Comp Neurol* **381**, 411-427 (1997).

13 Filippi, A., Mahler, J., Schweitzer, J. & Driever, W. Expression of the paralogous tyrosine hydroxylase encoding genes th1 and th2 reveals the full complement of dopaminergic and noradrenergic neurons in zebrafish larval and juvenile brain. *J Comp Neurol* **518**, 423-438 (2010). https://doi.org:10.1002/cne.22213

14 Yamamoto, K., Ruuskanen, J. O., Wullimann, M. F. & Vernier, P. Two tyrosine hydroxylase genes in vertebrates New dopaminergic territories revealed in the zebrafish brain. *Molecular and Cellular Neuroscience* **43**, 394-402 (2010). https://doi.org:10.1016/j.mcn.2010.01.006

15 Sur, A. *et al.* Single-cell analysis of shared signatures and transcriptional diversity during zebrafish development. *Dev Cell* **58** (2023). https://doi.org:10.1016/j.devcel.2023.11.001

16 Farrell, J. A. *et al.* Single-cell reconstruction of developmental trajectories during zebrafish embryogenesis. *Science* **360**, 979-+ (2018). https://doi.org:10.1126/science.aar3131

17 Raj, B. *et al.* Emergence of Neuronal Diversity during Vertebrate Brain Development. *Neuron* **108**, 1058-1074 e1056 (2020). https://doi.org:10.1016/j.neuron.2020.09.023

18 Hao, Y. *et al.* Integrated analysis of multimodal single-cell data. *Cell* **184**, 3573-3587 e3529 (2021). https://doi.org:10.1016/j.cell.2021.04.048

19 Raj, B. *et al.* Emergence of Neuronal Diversity during Vertebrate Brain Development. *Neuron* **108** (2020). https://doi.org:10.1016/j.neuron.2020.09.023
