# Supplementary material for: A genetic model for development, physiology and behavior of catecholamine-deficient zebrafish larvae
Source: Commun Biol. 2026 Aug 5;9:1045. doi: 10.1038/s42003-026-10732-9 (PMC13444079; doi:10.1038/s42003-026-10732-9)
Supplement: Supplementary file 2 [file 42003_2026_10732_MOESM4_ESM.pdf]

nature portfolio

Corresponding author(s): Wolfgang Driever

Last updated by author(s): Jun 29, 2026

Reporting Summary

Nature Portfolio wishes to improve the reproducibility of the work that we publish. This form provides structure for consistency and transparency in reporting. For further information on Nature Portfolio policies, see our Editorial Policies and the Editorial Policy Checklist.

Statistics

For all statistical analyses, confirm that the following items are present in the figure legend, table legend, main text, or Methods section.

n/a	Confirmed	
☐	☒	The exact sample size (*n*) for each experimental group/condition, given as a discrete number and unit of measurement
☐	☒	A statement on whether measurements were taken from distinct samples or whether the same sample was measured repeatedly
☐	☒	The statistical test(s) used AND whether they are one- or two-sided<br>*Only common tests should be described solely by name; describe more complex techniques in the Methods section.*
☒	☐	A description of all covariates tested
☐	☒	A description of any assumptions or corrections, such as tests of normality and adjustment for multiple comparisons
☐	☒	A full description of the statistical parameters including central tendency (e.g. means) or other basic estimates (e.g. regression coefficient) AND variation (e.g. standard deviation) or associated estimates of uncertainty (e.g. confidence intervals)
☐	☒	For null hypothesis testing, the test statistic (e.g. *F*, *t*, *r*) with confidence intervals, effect sizes, degrees of freedom and *P* value noted<br>*Give P values as exact values whenever suitable.*
☒	☐	For Bayesian analysis, information on the choice of priors and Markov chain Monte Carlo settings
☒	☐	For hierarchical and complex designs, identification of the appropriate level for tests and full reporting of outcomes
☐	☒	Estimates of effect sizes (e.g. Cohen's *d*, Pearson's *r*), indicating how they were calculated

Our web collection on statistics for biologists contains articles on many of the points above.

Software and code

Policy information about availability of computer code

Data collection

- ZEN (v3.4 blue edition) was used for imaging raw data and image processing
- Zeiss AxioVision SE64, Rel.4.9.1 for imaging of alkaline phosphatase-based in situ hybridizations
- IC Capture 2.4 software for recording heart function and optomotor response
- Noldus DanioVision (DVOC004x/T) for recording locomotor activity

Data analysis

- Ethovision XT14 for tracking locomotor activity
- Seurat package (v4.3.0) for transcriptomic analysis
- FFmpeg (v4.2.2 win64) for decompressing AVI recordings
- ImageJ (1.53c win64) for obtaining raw measurements coming from heart and optomotor response recordings
- OriginPro 2021 (64-bit, 9.8.0.200) for Fourier analysis of heart signals (according to Gaur et al., 2018)
- The R Foundation for Statistical Computing (R v4.2.3) was used for statistical analysis and graphs

For manuscripts utilizing custom algorithms or software that are central to the research but not yet described in published literature, software must be made available to editors and reviewers. We strongly encourage code deposition in a community repository (e.g. GitHub). See the Nature Portfolio guidelines for submitting code & software for further information.

Data

Policy information about availability of data

All manuscripts must include a data availability statement. This statement should provide the following information, where applicable:

- Accession codes, unique identifiers, or web links for publicly available datasets
- A description of any restrictions on data availability
- For clinical datasets or third party data, please ensure that the statement adheres to our policy

All numerical analyses of data are provided in the supplementary tables. All primary image data, videos and behavioral tracking are available on request.

Research involving human participants, their data, or biological material

Policy information about studies with human participants or human data. See also policy information about sex, gender (identity/presentation), and sexual orientation and race, ethnicity and racism.

Reporting on sex and gender	NA
Reporting on race, ethnicity, or other socially relevant groupings	NA
Population characteristics	NA
Recruitment	NA
Ethics oversight	NA

Note that full information on the approval of the study protocol must also be provided in the manuscript.

Field-specific reporting

Please select the one below that is the best fit for your research. If you are not sure, read the appropriate sections before making your selection.

☒ Life sciences ☐ Behavioural & social sciences ☐ Ecological, evolutionary & environmental sciences

For a reference copy of the document with all sections, see nature.com/documents/nr-reporting-summary-flat.pdf

Life sciences study design

All studies must disclose on these points even when the disclosure is negative.

Sample size	Sample sizes are indicated in Figures and Supplementary Tables
Data exclusions	Data points were not excluded for main figures. However, in order to reduce type II errors, an outlier analysis was carried out for optomotor response data. Results are attached as supplementary material
Replication	All experiments were carried out using biological replicates. Sample sizes are indicated in the figure legends and supplementary tables
Randomization	Figs. 3 - 6 samples were selected randomly. Figs. 7 - 10 samples were preselected for HGC phenotype & analysis in phenotypic categories was random.
Blinding	All experiments and analysis were performed blinded to genotype.

Behavioural & social sciences study design

All studies must disclose on these points even when the disclosure is negative.

Study description	NA
Research sample	NA
Sampling strategy	NA
Data collection	NA

Timing	NA
Data exclusions	NA
Non-participation	NA
Randomization	NA

Ecological, evolutionary & environmental sciences study design

All studies must disclose on these points even when the disclosure is negative.

Study description	NA
Research sample	NA
Sampling strategy	NA
Data collection	NA
Timing and spatial scale	NA
Data exclusions	NA
Reproducibility	NA
Randomization	NA
Blinding	NA

Did the study involve field work? ☐ Yes ☒ No

Field work, collection and transport

Field conditions	NA
Location	NA
Access & import/export	NA
Disturbance	NA

Reporting for specific materials, systems and methods

We require information from authors about some types of materials, experimental systems and methods used in many studies. Here, indicate whether each material, system or method listed is relevant to your study. If you are not sure if a list item applies to your research, read the appropriate section before selecting a response.

Materials & experimental systems

n/a	Involved in the study	
☐	☒	Antibodies
☒	☐	Eukaryotic cell lines
☒	☐	Palaeontology and archaeology
☐	☒	Animals and other organisms
☒	☐	Clinical data
☒	☐	Dual use research of concern
☒	☐	Plants

Methods

n/a	Involved in the study	
☒	☐	ChIP-seq
☒	☐	Flow cytometry
☒	☐	MRI-based neuroimaging

Antibodies

Antibodies used	1) Alexa Fluor®488 F(ab') fragment of goat anti-chicken IgG (A11039, Life Technologies)<br>2) Alexa Fluor®555 goat anti-mouse IgG (A21425, Life Technologies)<br>3) Anti-GFP, chicken IgY fraction, unconjugated (A10262, Life Technologies)<br>4) Anti-3A10, mouse IgG, supernatant (3A10-s, DSHB)<br>5) Anti-digoxygenin-alkaline-phosphatase (11093279910, Roche)<br>6) Rabbit anti TH (zebrafish) Ryu et al. (2007)
Validation	(1 - 5) Have been validated for Immunocytochemistry and Western Blot (website supplier).<br>6) Rabbit anti TH was validated in Ryu et al. (2007), Figure1

Animals and other research organisms

Policy information about studies involving animals; ARRIVE guidelines recommended for reporting animal research, and Sex and Gender in Research

Laboratory animals	Zebrafish (Danio rerio); Danio rerio, ABTL strain.<br>- Transgenic lines for observing catecholaminergic projections Tg(th:Gal4-VP16)m1233 and Tg(UAS:eGFP-CAAX)m1230 are described in Fernandes et al. (2012).<br>- Transgenic line for observing motor neurons Tg(mnx1:GFP)ml2Tg is described in Flanagan-Steet et al. (2005)<br>- Tyrosinase mutants tyr (allele tk20) are described in Page-McCaw et al. (2004).<br>- Tyrosine hydroxylase mutants th (allele m1403) and th2 (allele m1420) were generated in this work
Wild animals	The study did not involve wild animals
Reporting on sex	Sex is unknown, larvae are used before sex differentiation. Age is indicated in experiment descriptions
Field-collected samples	The study did not involve field-collected samples
Ethics oversight	Breeding and holding of zebrafish was approved by Regierungspraesidium Freiburg permit 35-9185.64/1.1;<br>All animal experiments were approved by state authorities (Regierungspraesidium Freiburg permits 35-9185.81/G-15/44 and 35-9185.81/G-16/96)

Note that full information on the approval of the study protocol must also be provided in the manuscript.

Plants

Seed stocks	NA
Novel plant genotypes	NA
Authentication	NA
